# Impact of Perineuronal Nets on Electrophysiology of Parvalbumin Interneurons, Principal Neurons, and Brain Oscillations: A Review

**DOI:** 10.3389/fnsyn.2021.673210

**Published:** 2021-05-10

**Authors:** Jereme C. Wingert, Barbara A. Sorg

**Affiliations:** Program in Neuroscience, Robert S. Dow Neurobiology Laboratories, Legacy Research Institute, Portland, OR, United States

**Keywords:** memory, perineuronal nets (PNNs), oscillations, parvalbumin, plasticity

## Abstract

Perineuronal nets (PNNs) are specialized extracellular matrix structures that surround specific neurons in the brain and spinal cord, appear during critical periods of development, and restrict plasticity during adulthood. Removal of PNNs can reinstate juvenile-like plasticity or, in cases of PNN removal during early developmental stages, PNN removal extends the critical plasticity period. PNNs surround mainly parvalbumin (PV)-containing, fast-spiking GABAergic interneurons in several brain regions. These inhibitory interneurons profoundly inhibit the network of surrounding neurons *via* their elaborate contacts with local pyramidal neurons, and they are key contributors to gamma oscillations generated across several brain regions. Among other functions, these gamma oscillations regulate plasticity associated with learning, decision making, attention, cognitive flexibility, and working memory. The detailed mechanisms by which PNN removal increases plasticity are only beginning to be understood. Here, we review the impact of PNN removal on several electrophysiological features of their underlying PV interneurons and nearby pyramidal neurons, including changes in intrinsic and synaptic membrane properties, brain oscillations, and how these changes may alter the integration of memory-related information. Additionally, we review how PNN removal affects plasticity-associated phenomena such as long-term potentiation (LTP), long-term depression (LTD), and paired-pulse ratio (PPR). The results are discussed in the context of the role of PV interneurons in circuit function and how PNN removal alters this function.

## Introduction

Perineuronal nets (PNNs) are specialized extracellular matrix structures surrounding specific neurons in the brain and spinal cord that appear during development in an experience–dependent manner (Dityatev et al., 2007; Balmer et al., 2009; Carulli et al., 2010) and restrict plasticity in adulthood. Although PNNs have been described as part of the extracellular reticulum for over 100 years by Golgi (see Celio et al., 1998), the seminal study that re-launched a precipitous increase in research on PNNs was a collaboration between Dr. James Fawcett, whose research focus was on the repair of spinal cord injury, and Dr. Tomasso Pizzorusso, whose focus was on critical period plasticity in the visual system. Their study revealed a major role for PNNs in the closure of the critical period for ocular dominance plasticity, and crucially, that this experience–dependent plasticity could be re-opened in adulthood by removing PNNs enzymatically (Pizzorusso et al., 2002). PNNs have now been shown to contribute to critical period plasticity within the visual, motor, and somatosensory systems (Pizzorusso et al., 2002; Barritt et al., 2006; Massey et al., 2006).

PNNs are found primarily around fast-spiking, parvalbumin (PV)-containing GABAergic interneurons within many brain regions (Härtig et al., 1992; Schüppel et al., 2002; Dityatev et al., 2007). However, PNNs also surround glutamatergic neurons (Wegner et al., 2003; Mészár et al., 2012; Horii-Hayashi et al., 2015; Vazquez-Sanroman et al., 2015; Yamada et al., 2015), which can be both PV positive or negative (Mészár et al., 2012; Horii-Hayashi et al., 2015), and other neurons involved in fast transmission, such as glycinergic output neurons in the medial nucleus of the trapezoid body (MNTB) at the calyx of Held synapse (Blosa et al., 2015; Balmer, 2016) and excitatory neurons in the deep cerebellar nucleus (DCN; Edamatsu et al., 2018; Hirono et al., 2018; Carulli et al., 2020).

Research from numerous laboratories demonstrate a central role for PNN-surrounded PV interneurons in plasticity, not only during the critical period, but also during learning and memory and repair of the damaged brain and spinal cord. Their dysfunction may contribute to a range of brain diseases/disorders, including schizophrenia, bipolar disorder, Alzheimer’s disease, autism spectrum disorder, epilepsy, and disorders associated with drugs of abuse and fear (Goldman-Rakic, 1994; Gogolla et al., 2009; Lewis, 2014; Rankin-Gee et al., 2015; Miyata and Kitagawa, 2016; Pantazopoulos and Berretta, 2016; Foscarin et al., 2017; Steullet et al., 2017; Fawcett et al., 2019; Testa et al., 2019). Removal of PNNs with the enzyme **chondroitinase ABC** (Ch-ABC) enhances reversal learning in the auditory cortex (Happel et al., 2014), promotes recovery of motor learning after spinal cord injury (Zhao and Fawcett, 2013) or cortical ischemia (Gherardini et al., 2015), and influences extinction of fear conditioning (Gogolla et al., 2009). Degradation of PNNs also modifies plasticity by strong stimuli: PNN degradation in the hippocampus, mPFC, or anterior cingulate cortex impairs reinstatement of fear conditioning (Hylin et al., 2013; Shi et al., 2019), and plasticity induced by cocaine or alcohol is impaired after PNN removal from the basolateral amygdala (BLA), insula, or mPFC (Xue et al., 2014; Slaker et al., 2015; Chen and Lasek, 2019). Removal of PNNs and/or the loose extracellular matrix thus provides an opportunity to alter plasticity for CNS repair after injury and to facilitate learning and memory in ageing and CNS disorders.

Although the past 20 years have brought about increased interest in research on PNNs and how they mediate brain plasticity, very little is still known about what form(s) this plasticity takes. How PNN removal impacts Hebbian activity-dependent plasticity mechanisms such as excitatory and inhibitory forms of spike-timing-dependent long-term potentiation (LTP) and long-term depression (LTD) on different cell types has also received relatively little investigation. PNN removal may also alter the intrinsic activity of neurons in addition to these other mechanisms. Perineuronal nets (PNNs) play such a critical role in PV interneuron function that recent cell-type specific manipulations have focused on manipulating PV interneurons alone to alter cortical plasticity (Cisneros-Franco and de Villers-Sidani, 2019). Therefore, we concentrate the first part of this review on the general properties and the wide range of circuit functions of PV interneurons, which are fast-spiking interneurons (Kawaguchi and Kubota, 1993) and make up the largest population of inhibitory interneurons in the cortex (Markram et al., 2004). We then discuss studies that examined the impact of removing PNNs or modifying PNN components on PV interneurons (Supplementary Table 1) and principal neurons that are generally, but not exclusively, glutamatergic pyramidal neurons (Supplementary Table 2). We then review findings from several studies that examine the impact of PNN removal on brain plasticity, including LTP, LTD, and paired-pulse ratio (PPR; Supplementary Table 3) followed by a discussion of how PNN removal or modifications impact brain oscillations, most notably gamma oscillations, in which PV interneurons play an integral role (Cardin et al., 2009; Sohal et al., 2009; Buzsaki and Wang, 2012; Supplementary Table 4). Finally, to bring together the broader implications of PNN removal on PV interneuron function, we discuss how PNN removal alters PV cell functionality in ways that may mediate changes in plasticity and memory-related phenomena. We have defined several terms in Box 1, which are presented in bold font. The reader is also referred to several additional reviews on PNNs (Sorg et al., 2016; Song and Dityatev, 2018; Bosiacki et al., 2019; Fawcett et al., 2019; Testa et al., 2019).

The vast majority of studies examining functional changes produced by PNN depletion have used Ch-ABC to degrade PNNs. Ch-ABC degrades chondroitin sulfate glycosaminoglycans on chondroitin sulfate proteoglycans (CSPGs; Yamagata et al., 1968) and hyaluronic acid (Galtrey et al., 2007). Some studies have used hyaluronidase, which degrades the hyaluronin backbone of PNNs, and a few studies have employed both Ch-ABC and hyaluronidase simultaneously. We also included studies in which key PNN components were genetically altered by knockout (KO) or knockdown strategies. We review studies mainly in mice and rats, in which most of the work has been conducted. With a few exceptions in which visual system critical period plasticity is included as a portion of the reviewed work (e.g., monocular deprivation studies), we have not focused on this literature and refer the reader to excellent primary literature and reviews of visual plasticity after PNN removal (Pizzorusso et al., 2002; Carulli et al., 2010; Beurdeley et al., 2012; Bernard and Prochiantz, 2016; Hensch and Quinlan, 2018; Reh et al., 2020).

Box 1 **Definitions**

- **Chondroitinase ABC (Ch-ABC)**: A bacterial enzyme derived from Proteus vulgaris that degrades chondroitin sulfates A, B, C, chondroitin sulfate, and hyaluronic acid (Yamagata et al., 1968).

**Inhibition:**

- **Blanket inhibition**: Inhibition resulting from the dense, non-specific connectivity of inhibitory neurons to the majority of the surrounding cell population (Karnani et al., 2014). Blanket inhibition does not posit that the strength of inhibitory contacts made by a cell are equal.
- **Feedback/recurrent inhibition**: Condition in which an excitatory cell activates an inhibitory cell that projects back to the initiating cell and inhibits it. Consequently, the pyramidal cell receives inhibitory feedback about the action potential it sent.
- **Feedforward inhibition**: Condition in which an excitatory input excites both an excitatory cell and an inhibitory cell (which inhibits the downstream excitatory cell) so that the compound signal is a monosynaptic excitatory input and a disynaptic inhibitory input with a delay.
- **Lateral inhibition**: Condition in a neuronal circuit in which an excitatory cell recruits an inhibitory cell that inhibits other nearby excitatory cells that may compete with other neurons to be the active members of the circuit.
- **Normalizing inhibition**: In its simplest form, normalizing inhibition is an extension of blanket inhibition but moves a step further by suggesting a functional role for interneurons to be recruited in a manner that is *scalable* with increases in circuit drive. As the number of active neurons in a cortical region increases, so, too, will the level of inhibition being provided to the neurons within that population (Murayama et al., 2009; Pouille et al., 2009).
- **Selective inhibition**: In contrast to blanket and normalizing inhibition, selective inhibition suggests that inhibitory cells receive specific input and will respond only to certain stimuli or input. Many studies suggest that inhibitory neurons often respond selectively to stimuli (Maurer et al., 2006; Runyan et al., 2010; Moore and Wehr, 2013; Lovett-Barron et al., 2014; Pinto and Dan, 2015; Allen et al., 2017; Najafi et al., 2020).
- **Shunting inhibition**: Local inhibition by GABA release onto a neuron whose membrane potential is near the inhibitory reversal potential. The result is a reduced magnitude of membrane potential change induced by nearby excitatory input.
- **Modulation**: Modulation describes a wide range of observations that suggest a neuron can change its responsiveness to inputs (responding with either more actions potential or fewer) without altering its selectivity to a particular stimulus. Common factors that impact modulation are movement, attention, neuromodulators, and other brain state changes (for an extensive review see Ferguson and Cardin, 2020).
- **Oscillations**: Neural population activity that has a stable rhythmic pattern of excitation and inhibition with a consistent temporal dynamic. Oscillations are typically measured with electrodes that record the summation of local neural activity.
- **Orthogonalization**: A form of pattern separation in which similar inputs are turned into near-mutually exclusive outputs (Wick et al., 2010). Feedforward excitation, feedforward inhibition, feedback inhibition, and lateral inhibition may support this in biological networks (Srivastava et al., 2008, 2014).
- **Pattern separation**: Pattern separation is the process of transforming inputs that may be highly similar into more distinct output patterns that would facilitate discrimination between similar events (for review see Cayco-Gajic and Silver, 2019).
- **Sparse encoding**: Sparse encoding simply posits that information is not being encoded by either a linear code (separate outputs for each relationship, i.e., grandmother cells where one cell represents each stimulus) or a dense code (every cell is active to some degree and the stimulus is represented by the whole population). In a sparse code, some number of cells (more than one cell and less than 100% of the population) may be active during any given stimulus.
- **Tuning (neuronal)**: When a neuron shows a specific response to a narrow range of presented stimuli. It is typically investigated in sensory modalities such as vision or hearing in which neurons respond to sweeps of stimuli, such as when black-and-white bars of varying orientations are presented to the eyes or tones of different frequencies are presented.

## Parvalbumin Interneurons: Circuits and Connectivity

### Role of PV Interneurons in Circuit Function

Parvalbumin interneurons are critical to neural circuit function. Recent advancements, including large neural population recording techniques and cell-type specific labeling and manipulation, have shown that PV interneurons play a major role in more complex neural circuit functions such as **modulation**, **sparse encoding** (or sparse coding), **pattern separation**, and interregional communication (for a more complete review of PV interneurons see Hu et al., 2014). How PNN removal impacts these circuit operations is largely unknown, although a growing body of literature suggests alterations in electrophysiological properties after their removal, which is expected to alter circuit properties. Thus, a fuller understanding of how PNN removal alters circuit functions would be highly informative for explaining plasticity events underlying behavioral changes.

Inhibitory circuit motifs, including **feedforward inhibition, feedback (recurrent) inhibition, and lateral inhibition** can greatly shape circuit responses to input. Parvalbumin neurons are involved in all of these types of inhibition, although the contribution of each of these types may be different depending on the region and/or cortical layer investigated (Pouille and Scanziani, 2001; Pouille et al., 2009; Espinoza et al., 2018). Parvalbumin neurons in both the cortex and the hippocampus form synapses primarily on the soma, axon initial segment, and distal dendrites, which allow these inhibitory neurons to effectively shunt their downstream targets and thereby control pyramidal cell output (Hu et al., 2014). Jouhanneau et al. (2018) found that single evoked or spontaneous action potentials in pyramidal cells can trigger unitary excitatory postsynaptic potentials (EPSPs) that reliably produce an action potential in a connected PV interneuron, which is not seen in monosynaptically connected somatosatin-expressing interneurons or pyramidal cells. This suggests that PV interneurons are particularly excitable during cortical Up states, such as during awake sensory processing, and that a single action potential efficiently recruits PV cell-mediated feedback inhibition, which reduces the probability of neighboring pyramidal cell activity for ~30 ms following the first pyramidal cell firing. Parvalbumin cells are highly excitable compared with other inhibitory neurons or principal cells, and they have higher mean firing rates (Avermann et al., 2012; Pala and Petersen, 2015). These properties suggest that PV interneurons may allow for synchronization of principal neuron output into neural assemblies, which are groups of neurons activated spatially and temporally to provide meaningful information (Badin et al., 2016). These properties also provide competitive inhibition to ensure that only the output cells receiving high enough drive can respond during states of inhibition.

To understand the role of PV interneurons at the circuit level, it is necessary to understand the typical inputs and outputs to and from these cells that might be indicative of their function. PV interneurons have long branching and radially projecting axons that form en-passant synapses, allowing for a large number of connections (Kisvárday et al., 2002; Packer and Yuste, 2011). Packer and Yuste (2011) used glutamate uncaging to activate specific PV interneurons while recording from pyramidal neurons. They tested the connectivity of an astounding 2,002 PV interneuron-to-pyramidal cell pairs in the mouse sensory and frontal cortex. Their results suggested that PV interneurons may form synapses non-specifically on nearly every principal cell within a 200 μm region (Packer and Yuste, 2011). High PV-to-pyramidal cell connection rates have been found in several other studies (Holmgren et al., 2003; Hofer et al., 2011; Espinoza et al., 2018; Jouhanneau et al., 2018). This widespread, non-specific inhibition is termed **blanket inhibition** (Karnani et al., 2014). In summary, PV interneurons are connected in all manners—associated with feedforward, feedback, and lateral inhibitory circuits. They have a high probability of connection with the cells in the neighboring 200 μm from their soma. This suggests that whether the input is coming locally or from a projection, PV interneurons will inhibit a *large proportion of the local population*. We discuss below what this excitability and connectivity suggest about functional roles for PV interneurons.

### Do PV Interneurons Produce Blanket Inhibition or Selective Inhibition?

Relevant to understanding PV cell function is the delineation of their inputs. Many investigators have suggested that PV interneurons represent a pool of inhibitory neurons that scale the amount of inhibition proportional to the amount of excitation (blanket inhibition). Thus, these neurons do not execute the complex computations as pyramidal cells do but instead sample cortical activity and linearly provide scalable blanket inhibition, termed **normalizing inhibition**, that dampens the response of pyramidal cells without altering their response properties (Atallah et al., 2012; Spanne and Jörntell, 2015; Trachtenberg, 2015). This suggests a high amount of convergence for PV cells to accurately sample and scale cortical inhibition.

The visual cortex is widely used to study neural connectivity, given the tuning properties of the neurons and the ability to map many features with different visual presentations in the same preparation. Many studies have looked at the tuning of visual cortex neurons to black-and-white gratings presented in various orientations in which many neurons increase their firing rate for specific orientations (orientation-selective neurons). In the rodent visual cortex, pyramidal neurons that show orientation tuning to different directions are scattered randomly across the cortex in what is described as a salt-and-pepper-like distribution (Ohki et al., 2005). In the visual cortex, PV interneurons have broad tuning where they fire similar numbers of action potentials for most orientations presented (Sohya et al., 2007; Niell and Stryker, 2008; Liu et al., 2009; Kerlin et al., 2010; Runyan et al., 2010; Hofer et al., 2011; Atallah et al., 2012; Wilson et al., 2012; Chen et al., 2013). One insightful study used serial electron microscopy in combination with two-photon *in vivo* calcium imaging to map pyramidal cell orientation responses and trace their proximal targets (Bock et al., 2011). They found a convergence of different orientation-selective neurons onto single inhibitory neurons in the mouse visual cortex. The broad tuning of many PV interneurons is therefore thought to reflect their local integration of pyramidal cell activity. This idea is further supported by a recent study on ocular dominance disparity in the binocular region of the mouse visual cortex (V1), where PV interneuron responses appear to be reflective of local aggregate population activity within a 100 μm area (Scholl et al., 2015). PV interneurons in the auditory cortex also appear to show a broad range of tuning to different frequencies (Moore and Wehr, 2013). The broad tuning of PV interneurons is also supported by a study in mouse visual cortex suggesting that PV interneurons lose direction selectivity in an activity-dependent manner following eye opening, suggesting that broad tuning is part of circuit maturation during development (Kuhlman et al., 2011). These studies provide evidence that PV interneurons often show broad **tuning** profiles due to nonselective input, and that, given their convergence onto many proximal excitatory cells, may indeed provide a blanket of inhibition based on the local integration of nearby activity.

However, all PV interneurons may not meet this function of broad selectivity. Many of the same studies that argue that PV interneurons have broad tuning profiles also showed narrow tuning profiles of PV interneurons in both the visual cortex and auditory cortex (Runyan et al., 2010; Wilson et al., 2012; Moore and Wehr, 2013). PV interneurons that show narrow tuning, likely tuning inherited from principal cells that have the same preference, would appear to be receiving specific input that would provide **selective inhibition**. These narrowly tuned PV interneurons suggest there are functional differences in *subsets* of PV interneurons in the visual system. One of these studies used calcium imaging to show that PV interneurons with narrow tuning in rodents do indeed have more neighboring pyramidal cells that share the same directional selectivity (Kerlin et al., 2010). Further support for this is suggested by hippocampal interneurons that showed spatial selectivity, which they appear to inherit from monosynaptically connected excitatory neurons (Maurer et al., 2006). English et al. (2017) also discovered that in the hippocampus, some fast-spiking neurons received a large number of presynaptic connections, making them suitable for scalable inhibition, while others showed fewer or more selective inputs, making them likely to be more selective. In the mouse posterior parietal cortex, inhibitory neurons are selective for choice, and their specificity is enhanced with learning alongside excitatory cells (Najafi et al., 2020). Classification of fast-spiking cells (PV-containing) in the cat visual system have yielded similar results, suggesting a distinction between narrowly tuned inhibitory cells vs. broadly tuned inhibitory cells and also suggest that there are differences in tuning selectivity between layers (Hirsch et al., 2003; Cardin et al., 2007; Nowak et al., 2008). Overall, it appears that many PV interneurons are involved in local integration and a scaling of inhibition based on local circuit activity, with some exceptions. Some PV interneurons do appear to be selective, although this may be due to anatomical location. Whether there are different roles for PV cells that are feature-selective vs. active over a broad range of inputs has yet to be seen. More work will be needed to determine if all PV interneurons play a role in blanket inhibition or if there are more complex computational roles played by inhibition.

### Role of PV Interneurons in Modulation of Principal Neuron Responses

Modulation is the change in the response profile of a neuron to input and is present in many circuits across the brain. Modulation shapes how neurons respond to stimuli depending on attention, arousal, stimulus characteristics, and neuromodulators (for an in-depth review on mechanisms of gain modulation in the cortex see Ferguson and Cardin, 2020). The role of GABAergic inhibition on modulation is complex, as this modulation alters the response profiles and activity levels of neurons under different behavioral states across the brain. Most of the studies discussed here have been done in the visual system, as neurons in the visual cortex show many forms of modulation, and the role of specific interneuron subtypes has been extensively studied. For example, a neuron in the visual cortex may respond maximally to a specific orientation of moving black-and-white patterned bars presented in front of the eyes, yet under certain situations, such as state of arousal or if a second stimulus is presented to a nearby part of the animal’s field of vision, the firing rate of the neuron responding to the same stimulus may increase or decrease. Modulation is typically investigated by generating input-output curves for a neuron (input = presynaptic neuron firing rate, injected current, stimulus intensity; output = firing rate). This is done under different conditions to determine the impact of those conditions on the new input-output curve. Modulation can be either multiplicative/divisive, called gain modulation or additive/subtractive. For gain modulation, the input or output axis of the response is transformed nonlinearly, changing the slope of the input/output curve; in other words, the magnitude of the modulation increases or decreases as a function of the input. For additive/subtractive modulation, the slope of the input-output curve is maintained but is shifted up, down, left, or right. These effects on input-output relationships likely depend on the complex interactions between different forms of inhibition and the statistics of the input, such as the degree of stochasticity.

Here, we will focus on the evidence that PV interneurons modulate the input-output relationship of principal neurons. Modeling suggests that **shunting inhibition**, typically attributed to PV interneurons given their synaptic inputs to the soma and proximal dendrites, would lead to subtractive modulation, which is best described by shifting the input-output response curve down the output axis (Ayaz and Chance, 2009). Optogenetic activation of PV interneurons in the visual cortex altered firing rate-current curves in a subtractive manner, while somatostatin cell activation led to a divisive modulation of principal neurons (Lee et al., 2012). These authors also found that PV interneuron activation decreased the tuning width (reduced the range of orientations near the preferred orientation that a neuron responds to) and increased the direction selectivity index (the responsiveness of the neuron to a drifting grating of a particular orientation was larger when the grating moved in a particular direction). This is in conflict with other reports suggesting that PV interneuron activation has a divisive gain modulation and that somatostatin neurons have a subtractive modulation on principal cell firing rate (Wilson et al., 2012). Ayaz and Chance (2009) reported that it may be difficult to discern the differences between these forms of modulation depending on stimulus parameters, noisy synaptic inputs near threshold, changes in response threshold, and response saturation. Indeed, in trying to reconcile the differences in outcomes of the above-mentioned studies, Lee et al. (2014) tested a wide range of laser stimulation protocols and found that varying levels of PV interneuron activation changed input-output curves and tuning width, with the strength of PV activation correlating with a sharpening of tuning selectivity. The authors concluded that PV interneuron activation results in subtractive modulation, fitting with models of PV shunting inhibition, but that the effect can appear to be divisive depending on the degree of PV activation. These studies suggest that PV interneuron activation can alter the input-output relationship of a neuron to the same stimuli and by doing so can *alter the selectivity of that neuron by rendering it responsive to a narrower range of input*.

### Role of PV Interneurons in Pattern Separation and Sparse Encoding

Exactly how information is encoded in the brain is still a matter of exploration and debate; however, there is evidence for some level of sparseness of representation (Spanne and Jörntell, 2015). In other words, at any given moment, the stimuli our brain receives and how it transforms these stimuli do not require the activity of the entire population of neurons, but activation is instead designated to a sparsely distributed population of neurons. This **sparse encoding** has many relevant implications for memory research, in that sparse encoding is a tradeoff between a local code, which is optimal for rapidly learning input-output assignments, and dense code, which is well suited to generalization (for review see Spanne and Jörntell, 2015). Related to sparse encoding is the concept of **orthogonalization**, which is often used to describe how a brain circuit encodes stimuli into non-overlapping representations. This is a form of **pattern separation**, which is the ability for a circuit presented with two similar yet non-identical inputs to separate these inputs into different patterns of activity, allowing for their disambiguation. It is theorized that a winner-take-all-mediated lateral inhibition potentially implemented by PV interneurons might be how the brain at least partially executes pattern separation and orthogonalization. In winner-take-all inhibition, a small number of principal cells that are preferentially recruited by the incoming input rapidly recruit inhibitory neurons that laterally project to and inhibit a separate competing assembly.

Parvalbumin interneurons appear to mediate the number of neurons that are allocated to a memory, and any deficits in either feedforward, feedback, or lateral inhibition would lead to a larger number of neurons incorporated into a memory (Morrison et al., 2016; Josselyn and Frankland, 2018). One example of this was demonstrated in a study in which optogenetic suppression of PV interneurons reduced the sparseness of neural activity in response to videos showing natural images in mouse V1 (Zhu et al., 2015). In this study, the authors showed that, given the patterns of cellular activity responding to certain frames of visual stimuli, it was possible to classify the frame that animals were being presented with a high degree of accuracy. When PV interneurons were suppressed, the classifier became less reliable at detecting the correct frame. This suggests that without PV interneuron activity, the neural response to dissimilar inputs, such as different frames, becomes more similar and harder to accurately decode. Therefore, decreasing PV cell activity increased the overlap of cellular activity in response to different inputs, and this overlap of activity makes it harder to discriminate what the input is based on neural activity alone. Another interesting study that illuminated the role of PV interneurons on neural synchrony and pattern separation used two-photon calcium imaging combined with optogenetic suppression of PV interneurons during spontaneous activity or visual orientation stimuli (Agetsuma et al., 2018). Suppression of PV interneurons increased activity, but it also increased the cell overlap for two stimuli that originally had a more separate representation in the brain (Agetsuma et al., 2018). PV interneuron-mediated winner-take-all lateral inhibition also appears to be supported as a mechanism for pattern separation in the dentate gyrus (Espinoza et al., 2018; Guzman et al., 2019; Braganza et al., 2020). Importantly, Braganza et al. (2020) suggested that a mechanism of pattern separation occurred in the dentate gyrus similar to that found in the cortex. They used a biologically constrained model of the dentate gyrus, wherein pattern separation can be precisely studied by examining the output of a network to many different patterns of input with varying degrees of overlap. Removing feedforward fast-spiking basket cell-mediated inhibition decreased pattern separation in their model. The higher probability of lateral inhibitory motifs in the dentate gyrus is thought to make it particularly important for pattern separation compared to other brain areas (Espinoza et al., 2018). It is clear that both feedforward and lateral inhibition indeed play roles in pattern separation (for a more complete review on pattern separation see Cayco-Gajic and Silver, 2019). In the ventral CA1, a subregion of the hippocampus important for social recognition, PV interneuron suppression decreased the ability of animals to discriminate between familiar and new animals (Deng et al., 2019). Altogether, these studies highlight a role for PV interneuron-mediated inhibition that allows for circuits to separate inputs and thus promote the successful encoding and retrieval of *different stimuli*.

Pattern separation is also dependent on the frequency of the input. For example, Braganza et al. (2020) found an enhanced degree of pattern separation for input coming in at the gamma vs. theta frequency range (discussed in more detail below). In addition, Jang et al. (2020) recently found that the activity of PV interneurons can gate the timing synchrony or coherence of cortical neurons. In this study, the authors recorded neural responses across all the layers of the barrel cortex and measured the level of synchrony across layers. By inhibiting or stimulating PV or somatostatin neurons optogenetically during whisker stimulation, they found that when whisker stimulation drove *low firing rate* responses in L4 input cells, PV interneurons synchronized activity across layers preferentially, but when whisker stimulation drove *high firing rate* responses in L4 input cells, PV interneurons desynchronized activity, producing sparser pyramidal cell activity. This finding suggests that PV interneuron-mediated feedforward inhibition preferentially synchronizes lower frequency input but desynchronizes higher frequency input. This desynchronization of activity is thought to be crucial to pattern separation (Cayco-Gajic and Silver, 2019). This suggests that PV-mediated desynchronization of principal cell activity is dependent on input frequency, and pattern separation may be optimal when input is in the high frequency range.

### Role of PV Interneurons in Brain Oscillations

Given their location around mainly fast-spiking interneurons, PNNs are in a prime position to alter the excitatory/inhibitory balance and thus regulate output. Several recent studies have focused on the role of PV interneurons in the generation of brain oscillations, including their role in generating the exquisite timing needed to fire action potentials during a specific phase of brain oscillations (Engel et al., 2001; Uhlhaas et al., 2010). PV interneurons may participate in many oscillations in the brain including theta, gamma, and sharp wave ripple activity (Klausberger et al., 2003; Cardin et al., 2009; Sohal et al., 2009; Stark et al., 2013; Schlingloff et al., 2014). For theta and gamma activity, this involvement in rhythmogenesis is further highlighted by both cellular and circuit-level resonant activity of PV interneurons at these frequencies (Pike et al., 2000; Cardin et al., 2009; Sohal et al., 2009; Sciamanna and Wilson, 2011; Stark et al., 2013; Moca et al., 2014; Ozawa et al., 2020). Fast oscillatory activity slowly increases in peak frequency during development and stabilizes in the gamma frequency range (30–80 Hz) during the fourth postnatal week in the mPFC, coinciding with the maturation of PV interneurons (Bitzenhofer et al., 2020). This entrainment of firing may also be further refined into adulthood; thus, a more finely tuned coupling occurs with PV cell maturation (de Almeida et al., 2013). Gamma, theta, and sharp-wave ripple activity all reflect rhythmic patterns of excitation and inhibition at different timescales (Fries et al., 2007; Buzsaki and Wang, 2012; Cardin, 2016; Sohal, 2016). In summary, PV interneurons have high excitability, provide widespread inhibition, and form circuit motifs that appear to be relevant in complex network activities such as circuit normalization, modulation, optimizing response profiles, and creating sparse network activity that may support pattern separation.

## Impact of PNN Depletion on PV and Principal Neurons, Plasticity, and Brain Oscillations

### Impact of PNN Depletion on Fast-Spiking (PV) Neurons

An early observation was that a key function of PNNs was to maintain the high frequency of firing in PV interneurons (Bruckner et al., 1993; Härtig et al., 1999). However, surprisingly few studies have examined the effects of PNN removal on intrinsic or synaptic properties of PNN-surrounded fast-spiking PV interneurons. Supplementary Table 1 shows a summary of the effects of PNN removal on fast-spiking interneurons, which are PV-containing neurons (Kawaguchi and Kubota, 1993). In these studies, PNN depletion was accomplished either by Ch-ABC treatment or by genetic modification of key PNN CSPGs *via* knockout or knockdown strategies. The majority of studies used mice and examined the hippocampus or visual cortex, and the most consistent change across all studies was the inability of fast-spiking (PV) neurons to maintain high firing frequencies after PNN removal.

#### Hippocampus

In hippocampal cultures, Ch-ABC decreased the firing threshold and afterhyperpolarization (AHP) of fast-spiking neurons, with no change in other intrinsic properties (Dityatev et al., 2007). Favuzzi et al. (2017) conducted an elaborate set of studies to examine the impact of manipulating a major PNN component, brevican (BCAN), on PV interneurons in hippocampal slices. In general, they found that the absence of BCAN in knockout (KO) mice or in mice with BCAN knockdown within PV interneurons had a decrease in the firing threshold, making PV interneurons more excitable, but with a decrease in the firing frequency and a decrease in the amplitude of the fast AHP (fAHP), the latter suggesting a decrease in gain. BCAN KO mice had a decreased frequency of both spontaneous excitatory postsynaptic currents (sEPSCs) and inhibitory postsynaptic currents (sIPSCs) as well as a decrease in mini-EPSCs (mEPSCs), with no changes in amplitude of sEPSCs, sIPSCs, mEPSCs, or mIPSCs. In general, these findings were supported by a decrease in the number of excitatory synaptic inputs apposing PV interneurons in both BCAN KO and BCAN knockdown mice, suggesting that BCAN contributes to the maturation of excitatory inputs. In the same studies, they also compared properties of PV interneurons surrounded by BCAN with PV interneurons naturally devoid of BCAN. They found several similar changes in the intrinsic and synaptic properties as listed above. The collective intrinsic properties support the ability of BCAN-surrounded PV interneurons to tune to higher firing frequencies with faster responses and, consistent with this, these neurons were surrounded by more excitatory puncta than were PV interneurons devoid of BCAN. Interestingly, they also demonstrated that BCAN knockdown decreased the clustering of Kv3.1b channels, which are critical for the fast-spiking nature of PV interneurons (Du et al., 1996; Goldberg et al., 2008), and in both BCAN KO and BCAN knockdown mice, there was a decrease in clustering of the AMPA receptor subunit GluA1. Altogether, these studies demonstrated mechanisms by which BCAN modifies tuning properties of PV interneurons to regulate cortical network activity. Hayani et al. (2018) examined the effects of short-term (2 h *in vitro*) and delayed (7 days *in vivo*) effects of Ch-ABC on fast-spiking interneurons in the mouse hippocampal CA2 region. Although they found no effects of acute Ch-ABC treatment, longer-term treatment reduced the firing threshold, decreased the frequency of sEPSCs and mEPSCs, and increased the frequency of sIPSCs, suggesting increased excitability of fast-spiking interneurons in the CA2 region, but possibly compensatory increases in inhibitory transmission to these neurons. Although a decrease in glutamatergic transmission is similar to the findings by Favuzzi et al. (2017) in the CA1 region with decreased synaptic glutamatergic input, Hayani et al. (2018) did not find an increase in glutamate puncta onto PV interneurons in the CA2 region.

#### Cortical Regions

Lensjø et al. (2017b) administered Ch-ABC in the visual cortex of awake adult rats and measured the activity of putative inhibitory neurons. Similar to the BCAN knockout discussed above, PNN removal decreased the mean spiking activity of fast-spiking interneurons. However, Ch-ABC also increased the spiking *variability* of these neurons, mimicking the firing during critical period plasticity in earlier developmental stages. They also reported decreased firing rates in inhibitory neurons, independent of whether the activity was spontaneous vs. visually-evoked, or whether rats were in an attentive vs. non-attentive state. Testing in a biologically-realistic network model of randomly connected excitatory and inhibitory neurons, the authors tested a range of excitatory and inhibitory synaptic efficacies and determined that shifts in the excitatory: inhibitory balance towards decreased inhibitory efficacy increased the spiking variability in both the excitatory and inhibitory populations, which supported the variability seen in their *in vivo* recordings. The authors concluded that the loss of inhibition and increased spiking variability represented an immature state of the excitatory: inhibitory network. This juvenile-like network state induced by Ch-ABC led to immediate increases in gamma activity after monocular deprivation (see “Impact of PNN Removal on Brain Oscillations” section). In a second study by the same group, and only the second study to examine *in vivo* putative inhibitory neurons following PNN removal, Christensen et al. (2021) found that Ch-ABC treatment in the medial entorhinal cortex produced a similar decrease in the mean firing rate of putative inhibitory neurons. This matched their findings in the visual cortex and suggests that PV interneurons have increased spiking variability and decreased spiking rates when measured *in vivo*. They additionally found that grid cell stability, which inhibitory activity is thought to maintain, is disrupted following Ch-ABC treatment. They matched their findings of grid cell instability with a computational model of grid cell dynamics by decreasing the excitatory drive onto inhibitory cells (matching the decreased firing rates seen *in vivo*). In addition, another study that used pharmacogenetics to suppress PV interneurons found similar network instability in the medial entorhinal cortex (Miao et al., 2017). Altogether, these studies provide strong evidence that PV cell activity is decreased following PNN removal *in vivo*.

In contrast to the findings from the rat visual cortex, in mouse visual cortical slices (V1, layer 4), Ch-ABC treatment in adults produced no changes in intrinsic properties of PV interneurons (Faini et al., 2018). However, Ch-ABC treatment increased the frequency and amplitude of sEPSCs and the frequency of mEPSCs, suggesting that excitatory thalamic inputs, which constitute the majority of synapses in layer 4 of the adult V1, are increased onto PV interneurons after PNN removal. This is similar to what they found in intact juvenile mice, indicating that Ch-ABC treatment transforms PV interneurons to a less mature state. No changes occurred in synaptic inhibitory inputs onto PV interneurons after Ch-ABC treatment, and the increase in sIPSC frequency and amplitude point to greater inhibition of the PV network. The authors concluded that Ch-ABC increased feed-forward inhibition by enhancement of glutamate transmission from the thalamus to PV interneurons, and that this may increase PV synaptic inhibition onto pyramidal neurons. They also tested changes in extracellular recorded potential amplitude to stimulation by black-and-white checkerboard patterns (drifting gratings) over a range of contrast values and found that Ch-ABC treatment reduced the amplitude of the response as contrast increased. Feedforward inhibition may have decreased the response of output neurons to higher contrast values as a function of gain, which provides additional evidence that Ch-ABC may indeed increase L4 feedforward inhibition. Miyata et al. (2018) used transgenic mice that overexpress chondroitin 6-sulfotransferase-1 (C6ST-1). The CSPG chains forming principal components of PNNs are disaccharide units composed of glucuronic acid and N-acetylgalctosamine (Sugahara and Mikami, 2007). These chains are sulfated at different positions, and the ratio of sulfation at the C4 and C6 positions changes in PNNs during development, with a high C6:C4 ratio in early development replaced by a low C6:C4 ratio in adulthood (Kitagawa et al., 1997). Thus, PNN content and, in turn, plasticity, could be reinstated by upregulating the C6ST-1 enzyme catalyzing C6 sulfation (Miyata et al., 2012). Transgenic mice overexpressing C6ST-1 showed reduced PNN formation in the visual cortex and maintained critical period plasticity into adulthood, which was accompanied by a decrease in resting membrane potential and an increase in action potential half-width in fast-spiking interneurons. These findings are consistent with those from Ch-ABC treatment, in which there is generally a reduced maturation and high plasticity state of PV interneurons.

Removal of PNNs by Ch-ABC in adult mouse somatosensory cortical slices (posterior medial barrel cortex) decreased the resting membrane potential and input resistance but did not alter the threshold or firing frequency of fast-spiking interneurons (Chu et al., 2018). However, this study did not examine current stimulation above 250 pA, and differences (perhaps decreased frequency; see above) may have been revealed at higher stimulation levels. Other measures of action potentials (half-width, amplitude) were reduced, suggesting a reduced excitability of these fast-spiking interneurons. Balmer (2016) treated somatosensory cortical slices of adult mice with Ch-ABC *in vitro* and reported no changes in intrinsic properties, but a decrease in the firing rate, a delay in firing, and lower gain using white noise current steps, indicating a decrease in the excitability of these fast-spiking neurons. This decreased excitability of PV interneurons may in turn impair the ability of their principal output neurons to appropriately filter incoming signals and in turn to fire reliably. Additionally, Tewari et al. (2018) found in the mouse somatosensory cortex that tumor development led to an increase in endogenous enzymes that remove PNNs. PV interneurons near the tumor had decreased PNNs that in turn increased cell capacitance, firing rates, and produced a more depolarized resting membrane potential. In control non-tumor slices of somatosensory cortex, pretreatment with Ch-ABC or infusion of Ch-ABC during recording captured the same changes. Together these results suggest that PNN removal may increase capacitance and therefore decrease excitability, which was further verified with a computational model.

**Figure 1:**
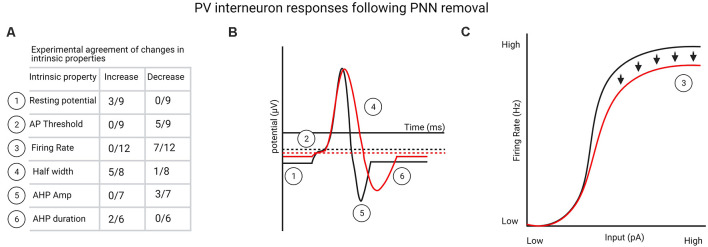
**(A)** List of perineuronal net (PNN) removal-mediated changes in parvalbumin (PV) interneuron intrinsic properties that one-third or more of the experiments agreed upon (see Supplementary Table 1 for a complete list of studies). **(B)** Graphical representation from table shown in **(A)** of changes in PV interneuron intrinsic properties following PNN removal. **(C)** Experimentally agreed upon firing rate changes to PV interneurons following PNN removal, indicating deficits in firing rate at high stimulation levels. Created with BioRender.com.

Overall, it is apparent from these relatively few studies that PNN degradation produces a variety of electrophysiological responses in fast-spiking PV interneurons, but in most cases, PNN removal appears to reduce the function of PV interneurons and return them to a juvenile-like, less mature state. In particular, one-third to more than half the studies found: (1) an increase in resting membrane potential; (2) a decrease in the firing threshold; (3) a decrease in firing rate; (4) an increase in action potential half-width; (5) a decrease in AHP amplitude; and (6) an increase in AHP duration (Figures 1A–C). However, both local effects and network effects need to be considered, given that PV interneurons in different layers receive different levels of thalamic input (L4 vs. L2/3/5) and, as a result, may vary in their response properties and serve different functions across circuits (Cardin et al., 2007; Faini et al., 2018). The response to PNN removal is likely to depend on the brain region and circuit-level differences as well as the interval over which Ch-ABC- or hyaluronidase-induced circuit changes occur before examining electrophysiological properties. Moreover, some differences may depend on whether PNNs are allowed to develop normally until adulthood and whether PNN removal is conducted *in vitro* vs. *in vivo*. In summary, due to the limited number of studies that directly tested the impact of PNN removal on fast-spiking PV interneurons, it is difficult to construct a fully cohesive picture of the mechanisms by which PV interneuron function is altered or transitioned to a less mature state. Future studies focused on assessing *in vivo* functioning of PV interneurons would greatly help delineate unitary and network properties in intact systems.

### Impact of PNN Depletion on Principal Neurons

Supplementary Table 2 shows a summary of the findings from PNN depletion on principal output neurons, most of which have measured glutamatergic neurons in either the hippocampus or cortex. However, other studies have examined output neurons that themselves are surrounded by PNNs and are involved in fast signaling, such as glycinergic neurons in the medial nucleus of the trapezoid body (MNTB) at the calyx of Held synapse and glutamateric neurons in the deep cerebellar nuclei (DCN) onto which cerebellar Purkinje cells form synapses. In general, PNN removal produced few changes in electrophysiological properties of principal neurons, especially those neurons not surrounded by PNNs.

#### Hippocampal Cultures

Several studies have examined the effect of PNN depletion *in vitro* on cultured hippocampal neurons or neurons co-cultured with astrocytes (Dityatev et al., 2007; Frischknecht et al., 2009; Pyka et al., 2011; Orlando et al., 2012; Geissler et al., 2013). Dityatev et al. (2007) found that treatment of cultures with Ch-ABC for 48 h did not alter intrinsic properties of principal (pyramidal) neurons and, consistent with this finding, Frischknecht et al. (2009) used cultured hippocampal neurons, in which pyramidal neurons become surrounded by BCAN after 3 weeks. They reported that treatment with hyaluronidase for 24 h produced no changes in intrinsic properties of principal neurons or altered excitatory synaptic input (mEPSCs). However, hyaluronidase increased the diffusion of α-amino-3-hydroxy-5-methyl-4-isoxazolepropionic acid receptor (AMPARs). Interestingly, a follow-up study did not find an effect of hyaluronidase in AMPAR diffusion in *aspiny interneurons* (Klueva et al., 2014). Pyka et al. (2011) measured principal neurons in hippocampal cultures that were exposed to Ch-ABC for 13 days. They observed no differences in sodium-or potassium-activated currents or in mIPSCs but did find a decrease in the amplitude of mEPSCs, consistent with the idea that AMPARs diffuse laterally in the absence of PNNs (Frischknecht et al., 2009). Orlando et al. (2012) recorded mEPSCs in CA1 neurons after Ch-ABC treatment in culture; these were not altered despite an increased remodeling of synaptic spines. Importantly, however this spine remodeling was examined far from where PNNs were located (cell body and proximal dendrites), indicating that CSPG removal by Ch-ABC was sufficient for this remodeling to occur. Geissler et al. (2013) tested the impact of quadruple knockout of ECM proteins that serve as major components of the PNN during development and/or adulthood. Knockout mice lacking tenascin-C (TN-C), tenascin-R (TN-R), BCAN, and neurocan within neurons, astrocytes, or both, had reduced PNNs as expected and reduced excitatory and inhibitory inputs by 3 weeks in culture. These inputs were reflected in decreases in the frequency of mIPSCs and mEPSCs. It is difficult to draw definitive overall conclusions from these studies in cultured cells, given the heterogeneity of treatments and preparations. Nonetheless, PNN removal alters synaptic changes to cultured neurons, and in some cases, their postsynaptic responsiveness to inputs. Also important to bear in mind is that several changes mediated by the loss of PNN components by Ch-ABC treatment may be attributed to loss of the diffuse ECM present throughout dendritic arbors.

#### Hippocampal Slices: CA1, CA2, Ventral Hippocampus

Studies in slices of hippocampal CA1 neurons have tested the effects of PNN alterations on properties of principal neurons using *in vitro* Ch-ABC (Khoo et al., 2019) *in vivo* Ch-ABC, an shRNA to decrease BCAN levels in the CA1 region (Shi et al., 2019), TN-R KO mice (Saghatelyan et al., 2001), BCAN KO mice (Brakebusch et al., 2002), or hyaluronidase (Kochlamazashvili et al., 2010). Khoo et al. (2019) found no changes in the firing rate of CA1 neurons but that the ratio of EPSPs to IPSPs increased in CA1 principal neurons, and this increase was attributed to a decrease in stimulated IPSP amplitude. The increased excitatory: inhibitory ratio was possibly produced by decreased GABA transmission from PV interneurons, as mIPSC frequency was also reduced after Ch-ABC treatment. Shi et al. (2019) delivered Ch-ABC into the mouse CA1 region for 24 h and found increases in the frequency and amplitude of sIPSCs and amplitude of mIPSCs. In the same set of studies, brevican shRNA in the CA1 produced some similar effects, and overexpression of a key link protein in PNNs, HAPLN-1, had opposing effects on the frequency of sIPSCs and mIPSCs, overall suggesting that PNN removal increased GABAergic transmission in the CA1. This result is opposite to the *decreased* GABA transmission reported by Khoo et al. (2019) and may be attributed to the time and route of Ch-ABC treatment (2 h *in vitro* vs. 24 h *in vivo*), but the mechanisms mediating these opposing effects on CA1 pyramidal neurons remains unknown. Genetic knockout of BCAN did not alter sIPSCs, unlike the increases in sIPSC amplitude and frequency in mice given BCAN shRNA, suggesting that compensatory effects may have occurred during development in these KO mice. Genetic knockout of TN-R produced an increase in the frequency of mEPSCs and mIPSCs, and increased the amplitude of the periosomatic unitary current, suggesting that there was decreased inhibition of pyramidal neurons in TN-R KO mice. Hyaluronidase treatment did not affect intrinsic properties of CA1 neurons (Kochlamazashvili et al., 2010).

Degradation of PNNs *in vivo* has also been examined in the CA2 region in hippocampal slices from juvenile mice (Carstens et al., 2016) and young adults (Hayani et al., 2018). The mouse CA2 region is enveloped in a dense meshwork of PNNs and ECM that binds to *Wisteria floribunda* agglutinin (WFA), a marker for PNNs. While many of the PNNs surround inhibitory neurons in the CA2 of mouse [and fewer in the rat (Lensjø et al., 2017a)], PNNs also surround excitatory neurons in this region (Celio, 1993; Carstens et al., 2016; Lensjø et al., 2017a). In contrast to the CA1 region, synaptic plasticity is relatively low in projections from the CA3 to the CA2 (Zhao et al., 2007; Chevaleyre and Siegelbaum, 2010). Carstens et al. (2016) found that Ch-ABC treatment for 2 h did not alter intrinsic properties of CA2 neurons, with exception of a decrease in input resistance. However, in contrast to the inhibitory effects of Ch-ABC on LTP in the CA1 region discussed above, Ch-ABC exposure *increased* LTP in the CA2 region. Thus, PNNs appear to play a role in preventing plasticity of the CA3 to CA2 pathway. A second study examined both acute (2 h) and longer-term (7 days), Ch-ABC treatment in the CA2 region of 3–5 week old mice (Hayani et al., 2018) and found only minor changes in principal neurons. There were no *acute* effects of Ch-ABC on intrinsic or synaptic properties, but *longer-term* Ch-ABC treatment rendered principal CA2 neurons more excitable by decreasing the latency of action potential firing and increasing the decay time of sIPSCs, consistent with decreased GABA input or responses to inhibitory input. This study in particular reveals the importance of examining *time-dependent modifications* in the plasticity of synaptic and network properties after PNN degradation. Shah and Lodge (2013) conducted *in vivo* electrophysiology in the ventral hippocampus of anesthetized adult mice to assess the impact of longer-term Ch-ABC treatment (7 days) on pyramidal neurons. They found an increase in the firing frequency of pyramidal neurons, which is consistent with the reduced function of PNN-surrounded interneurons after Ch-ABC treatment.

#### Visual Cortex

Faini et al. (2018) conducted slice electrophysiology in the mouse adult visual cortex (V1) to measure the effect of Ch-ABC (2–3 days) treatment on principal neurons. They measured L4 neurons, the majority of which receive glutamatergic input from the thalamus. Similar to other studies, there were no changes in intrinsic properties and no changes in sEPSCs or sIPSCs. However, they found an increase in the amplitude of visual stimulus-evoked IPSCs in principal neurons when stimulating at above-threshold levels the thalamic inputs that synapse onto PV interneurons, indicating higher inhibition of principal neurons. Monocular deprivation reduced this effect of Ch-ABC, showing that Ch-ABC effects were dependent on incoming visual stimuli. Overall, they found enhanced feedforward inhibition after Ch-ABC, but the effects of Ch-ABC were less impactful on principal neurons than on PV interneurons. Lensjø et al. (2017b) used *in vivo* electrophysiology in the visual cortex (V1) of awake adult rats to examine how Ch-ABC given 3–14 days earlier affected putative excitatory neurons. They found no change in the firing rate of excitatory output neurons, but, similar to their findings in inhibitory neurons, there was increased spiking *variability*. This variability in both excitatory and inhibitory neurons suggested to the authors that Ch-ABC decreased the stability of the excitatory: inhibitory network, which they attributed to a decrease in inhibition by fast-spiking PV interneurons (see above).

#### Medial Nucleus of the Trapezoid Body (MNTB)

Two studies examined the impact of reducing PNNs on the principal (glycinergic) medial nucleus of the trapezoid body (MNTB) neurons. Blosa et al. (2015) used *in vivo* recordings in BCAN KO mice and found decreases in the firing rate evoked by sound, with a broader presynaptic action potential and a broader post-synaptic EPSP. Together with reduced vGlut1 in calyx terminals in the MNTB, these findings suggested a decrease in glutamate release at the calyx of Held synapse in BCAN KO mice. Consistent with the decreased response to sound in BCAN KO mice, Balmer (2016) delivered Ch-ABC in slices containing MNTB neurons and also found that these neurons fired less to white noise current, with no changes in intrinsic properties. In addition, Ch-ABC increased the amount of current needed for MNTB neurons to fire an action potential. The voltage threshold was more depolarized, but spike amplitude and acceleration of membrane potential changes after stimulation were lower. The authors concluded that intact PNNs allowed for an increased gain of MNTB neurons in response to incoming stimuli.

#### Cerebellum

In the cerebellum, Edamatsu et al. (2018) reduced PNNs by knockout of HAPLN2 (BRAL2), one of the main link proteins contributing to PNN development in the cerebellum (Carulli et al., 2010; Bekku et al., 2012). They examined two main synapses: those from inhibitory Purkinje neurons to deep cerebellar nuclei (DCN) principal neurons, and those from excitatory neurons originating from mossy and climbing fibers. They found a decrease in IPSCs evoked from Purkinje cell stimulation without changes in amplitude, suggesting that the absence of PNNs reduced the number of GABA terminals. Hirono et al. (2018) used Ch-ABC in acute slices containing DCN neurons and found a decrease in sIPSCs and mIPSCs, suggesting that these changes may mediate the greater learning (eyeblink conditioning) they observed in mice treated with Ch-ABC; this learning is dependent on Purkinje outputs to large DCN neurons. Consistent with their findings supporting decreased inhibitory transmission on DCN neurons, using a lentivirus approach to chronically release Ch-ABC into the DCN, Carulli et al. (2020) demonstrated a reduction in spontaneous activity of DCN neurons after Ch-ABC treatment. They speculated that this reduction was due to increased inhibitory and decreased excitatory input onto these neurons and could underlie their observation of enhanced plasticity during the acquisition of eyeblink conditioning but poorer retention of this memory.

#### Somatosensory Cortex and Medial Prefrontal Cortex

In barrel field cortical slices, Chu et al. (2018) treated slices from the somatosensory cortex (post barrel medial subfield) with Ch-ABC in juvenile mice, and reported no changes in intrinsic properties or in sEPSCs of regular-spiking neurons. Slaker et al. (2015) examined *in vivo* Ch-ABC treatment effects on excitatory output neurons in slices from the rat medial prefrontal cortex (mPFC) 9 days later. They found an increase in the firing rate of these neurons, with a decrease in mIPSC frequency and no changes in mIPSC amplitude. The mIPSC frequency trended toward a decrease when examined 3 days later (unpublished observations), indicating that the outcome on synaptic properties likely depended on the interval of time after Ch-ABC treatment. Overall, the findings in the mPFC are in accordance with others demonstrating a decrease in inhibitory input onto these neurons after Ch-ABC treatment. Tewari et al. (2018) measured the impact of PNN removal by the endogenous release of enzymes following tumor development in the somatosensory cortex and measured changes in pyramidal cells. They also measured changes due to acute PNN removal by Ch-ABC in control slices of the somatosensory cortex. They found that PNN removal due to endogenous enzymes near the tumor depolarized the resting membrane potential and lowered the current threshold for AP initiation. This increased the firing frequency of pyramidal cells. Acute PNN removal caused only a slight increase in firing rate, leaving other intrinsic properties unaltered. This adds evidence suggesting that, overall, many intrinsic properties of pyramidal neurons are unchanged following acute PNN removal but, in some cases, these output neurons show slightly increased firing rates.

In general, an impact of PNN removal on electrophysiological properties is most often found within the cells enwrapped by PNNs, whether they are fast-spiking, PV-containing interneurons or principal neurons (the latter being MNTB glycinergic or hippocampal CA2 glutamatergic neurons). However, it must be kept in mind that the majority of studies have examined the influence of PNN removal after short intervals, whereas a reorganization of the network in addition to intrinsic and synaptic consequences may require days rather than hours (Hayani et al., 2018).

### Impact of PNN Removal on Synaptic Plasticity

Several studies focused on the effects of PNN removal on plasticity events within principal neurons, including LTP, LTD, and PPR, and these are separately grouped in Supplementary Table 3 and include some of the studies briefly discussed above. The majority of studies examining how PNNs altered brain plasticity in adults tested the impact of either Ch-ABC or the genetic knockout of key PNN components on LTP or LTD in the hippocampus.

#### LTP

Seven studies examined LTP in hippocampal CA1 slices after stimulation of Schaffer collaterals. Six of these found a decrease in LTP after Ch-ABC or hyaluronidase (Bukalo et al., 2001; Kochlamazashvili et al., 2010; Shi et al., 2019), after shBCAN treatment (Shi et al., 2019), or in BCAN, TN-R, or neurocan KO mice (Bukalo et al., 2001; Saghatelyan et al., 2001; Zhou et al., 2001; Brakebusch et al., 2002). Importantly, one study found that Ch-ABC treatment did not *further* alter LTP in TN-R KO mice (Bukalo et al., 2001). In another study (Shi et al., 2019), the effect of Ch-ABC was blocked by picrotoxin and, interestingly, Ch-ABC treatment in the CA1 caused an LTD induced by theta burst stimulation in the presence of glutamate receptor antagonists to switch to LTP. Contrary to the findings of reduced inhibition after Ch-ABC removal (e.g., Lensjø et al., 2017b), the authors suggested that PNNs limited feedback inhibition by PV interneurons onto CA1 principal neurons, whereas PNN removal produced greater inhibition to these neurons to prevent LTP maintenance. In contrast to the findings above showing a decrease in LTP after PNN removal, Riga et al. (2017) did not find that Ch-ABC altered LTP in the CA1, but this was tested 12–24 days after *in vivo* Ch-ABC treatment, a time when PNNs appeared to be partially or largely restored. Bikbaev et al. (2015) used hippocampal cultures grown on microelectrode arrays to examine the effect of hyaluronidase on network activity. They first determined that the bursting activity of populations (multiple neurons measured simultaneously) correlated with maturation of the ECM. Acute hyaluronidase added to matured cultured neurons (4 weeks) increased the spiking and bursting rate, thus disinhibiting the network.

**Figure 2:**
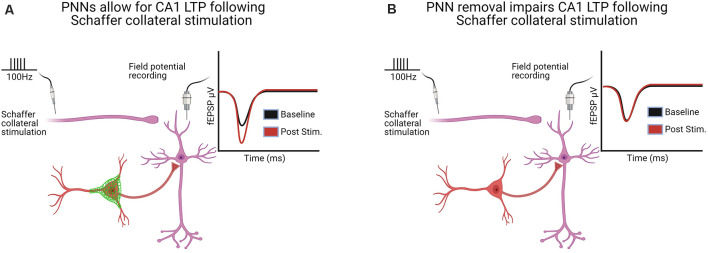
Hippocampal long-term potentiation (LTP) in the CA1 following Schaffer collateral stimulation was altered in six of the seven studies following PNN removal [see Supplementary Table 3 for a complete list of LTP/long-term depression (LTD) and paired-pulse ratio (PPR) studies]. **(A)** With PNNs intact, Schaffer collateral stimulation induces LTP. **(B)** With PNNs removed, Schaffer collateral stimulation-induced LTP is impaired. Created with BioRender.com.

Jansen et al. (2017) used quadruple knockout mice deficient in TN-C, TN-R, BCAN, and neurocan to examine LTP in the dentate gyrus of awake mice. They found a bidirectional impact on LTP, wherein high-frequency stimulation produced an initial decrease in field EPSPs (fEPSPs) followed by a delayed LTP, with no differences when a weak stimulation was used, but the resulting LTP was NMDA dependent only in control mice. A follow-up study by this group examined in mouse hippocampal multielectrode arrays how the quadruple knockout within either neurons or astrocytes altered the spontaneous activity of the network (Gottschling et al., 2019). Their studies revealed an enhanced network activity, consistent with their previous work demonstrating enhanced LTD in the dentate gyrus of awake KO mice discussed above (Jansen et al., 2017). It is not apparent how the increased network activity is in accordance with their studies demonstrating a decrease in both mIPSCs and mEPSCs; however, it is important to keep in mind that several genes are altered in quadruple knockout mice (Jansen et al., 2017; Gottschling et al., 2019), and thus the mechanisms underlying LTP may be different. Overall, the majority of studies show that PNN removal decreases LTP, while no studies showed an increase in LTP. This decrease in LTP is depicted in Figures 2A,B.

#### LTD

Fewer studies have examined the impact of PNN removal on LTD, and the effects are less consistent than for LTP, with both increases and decreases found. Khoo et al. (2019) found that Ch-ABC increased LTD in the CA1 of adult mice, similar to what they found in young mice prior to PNN development, and this effect was dependent on NMDA and GABA receptor-mediated effects. In contrast, Bukalo et al. (2001) reported that, while short-term depression was not altered after Ch-ABC treatment, LTD was reduced by this treatment in the CA1 region (with no change in TN-R KO mice). Khoo et al. (2019) attributed the differences between these opposing findings to the stimulation parameters used to induce LTD, but the mechanisms underlying downstream events from different stimulation parameters leading to LTD remain to be determined. Ch-ABC blocked a GABA-mediated LTD in the hippocampal CA1 region (Shi et al., 2019). In the hippocampal dentate gyrus, Jansen et al. (2017) did not observe LTD but did elicit short-term depression that was larger and longer-lasting in quadruple KO mice for TN-C, TN-R, BCAN, and neurocan, suggesting higher plasticity in the absence of these proteins.

In the perirhinal cortex, Ch-ABC treatment increased object recognition memory and LTD (Romberg et al., 2013), which is needed for this memory task (Griffiths et al., 2008). In the same study, similar effects on LTD and behavior were found in KO mice deficient in the PNN component Crtl-1 (also known as HAPLN1). The increase in basal synaptic transmission and failure to induce paired-pulse facilitation (PPF; see below) in both Ch-ABC and Crtl-1 KO mice indicated a shift to enhanced plasticity that mediated the longer-term memory in this task, but the mechanism remains to be determined.

#### Paired-Pulse Ratio (PPR)

Supplementary Table 3 also shows the results from several studies that measured the PPR to assess whether changes in short-term synaptic plasticity occurred after PNN manipulation. The majority of studies were conducted in the hippocampus CA1 or CA2 regions. Most studies demonstrated no change in PPR after treatment with either Ch-ABC (Bukalo et al., 2001; Carstens et al., 2016; Khoo et al., 2019; Shi et al., 2019), hyaluronidase (Kochlamazashvili et al., 2010), after knockdown of BCAN using an shRNA (Shi et al., 2019), or in BCAN, neurocan, or TN-R KO mice (Bukalo et al., 2001; Saghatelyan et al., 2001; Zhou et al., 2001; Brakebusch et al., 2002). Only one study demonstrated an increase in the PPR [decrease in hippocampal paired-pulse depression (PPD); Frischknecht et al., 2009] after hyaluronidase treatment, which was further shown to be influenced by the ability of glutamate-induced currents to produce AMPAR diffusion and replace rapidly desensitized AMPARs. The difference in PPR among studies in the hippocampus may be attributed to the fact that this latter study was the only one that employed cultured neurons, and thus, circuit formation is expected to differ from that in hippocampal slices. An increase in PPD of evoked IPSCs has also been found after Ch-ABC treatment in cerebellar DCN neurons (Hirono et al., 2018), while no change was observed in these neurons in knockout mice deficient in a major link protein, Bral2 (Edamatsu et al., 2018). In the perirhinal cortex, both Ch-ABC and link protein Crlt1 KO mice demonstrated a reduced PPR (Romberg et al., 2013). Overall, this form of short-term plasticity was rarely found after PNN manipulation, with only a few regions demonstrating changes.

### Impact of PNN Removal on Brain Oscillations

#### Gamma Oscillations

Supplementary Table 4 shows the 11 studies to date that have investigated synchronous neural activity while manipulating PNNs. Of these studies, six specifically interrogated spectral power changes in the gamma frequency range (>30 Hz). Two studies from the same group, Cabungcal et al. (2013) and Steullet et al. (2014), found in anterior cingulate cortical slices that application of a mixture of carbechol, kainate, and quinpirole increased the power of high-frequency beta and gamma range oscillatory activity after Ch-ABC treatment. It should be noted that in these studies, power was measured after high-frequency oscillations became stable, which is not typical of *in vivo* high-frequency oscillations that are only transient in nature. This work suggests that PNN removal may increase the ability for a strongly driven circuit to resonate at the high frequency range, with potentially higher synchrony, given the increase in power. Gurevicius et al. (2004) also found an increase in both hippocampal and cortical EEG amplitude for frequencies over a broad gamma range as well as an increase in the amplitude of auditory-related event potentials. The authors attributed the increase in local field potential power to a decrease in perisomatic inhibition. In another *in vivo* study in the rat visual cortex, Lensjø et al. (2017b) also found increases in spontaneous gamma power prior to visual stimulus onset, but no differences in evoked power to drifting grating stimuli in awake, freely-moving animals treated with Ch-ABC. No changes in the broad or narrow gamma power, number of gamma events (periods of time where power exceeded a certain threshold), or mean power of gamma frequency events, were different during visually evoked activity. They also noted an increase in gamma power in the hour following monocular deprivation in Ch-ABC treated animals. Faini et al. (2018) reported an increase in both spontaneous and visually evoked power over a broad gamma frequency range. Carceller et al. (2020) found that anesthetized animals treated with Ch-ABC 4 days prior had decreased power over a broad spectrum, with significant decreases in high gamma (70–100 Hz) and fast gamma (>100 Hz) following tail pinches. They found no difference after Ch-ABC treatment in phase amplitude coupling, in this case, modulation of high and fast gamma power depending on the local theta phase. Differences in task (spontaneous vs. evoked, anesthetized vs. awake), PNN removal (Ch-ABC volume and time of treatment vs. specific component manipulations), and brain regions all make it difficult to define specific changes in gamma oscillations following PNN removal.

Several studies also examined the impact of direct manipulation of PV interneurons on gamma oscillations. Optogenetic activation of PV interneurons is known to create stable network oscillations in a narrow gamma frequency range ~40 Hz (Cardin et al., 2009; Sohal et al., 2009; Chen et al., 2017). Inhibition of PV interneurons has been shown to cause both increases in spectral power over a broad gamma frequency range and decreases in narrowband gamma activity (Cho et al., 2015; Chen et al., 2017; Abbas et al., 2018; Sohal and Rubenstein, 2019; Guyon et al., 2021). These findings are seemingly contradictory, as both activation and inhibition of PV interneurons can cause gamma power activity. Some observed discrepancies are likely due to differences in recording preparations in which optogenetic suppression of PV interneurons in anesthetized animals (Sohal et al., 2009) may decrease gamma power, whereas PV suppression in awake and behaving animals may show different changes in the power spectrum that depend on the behavior. Overall, these results are consistent with the idea that decreased inhibitory function can lead to broadband increases in spontaneous gamma power but deficits in narrowband, evoked gamma oscillatory activity (Cho et al., 2015; Sohal and Rubenstein, 2019; Guyon et al., 2021). While a few studies show increases in gamma power after PNN removal, it is still unclear whether increases are reflective of aberrant network instability or true oscillations.

#### Theta Oscillations

As discussed earlier, PV interneurons are linked to theta frequency oscillations, in which they show a high degree of phase-locking and provide a source of inhibition that can induce theta resonant membrane oscillations in pyramidal cells (Stark et al., 2013). Therefore, like gamma oscillations, disruptions to PNNs may cause alterations in theta rhythms. Hippocampal local field potential oscillations in the 4–12 Hz range are a prominent feature during periods of mobility. Gurevicius et al. (2004) found that in TN-R knockout mice, the theta power was not altered during movement, but the peak of the frequency was shifted lower. They also found that spontaneous cortical theta power was increased during periods of immobility. Christensen et al. (2021) found in freely-moving rats that Ch-ABC treatment decreased the theta peak frequency and increased theta power in the medial entorhinal cortex, a structure that, like the hippocampus, shows modulation of theta power driven by movement. These authors also found an increase in theta power in the hippocampus, even though Ch-ABC treatment was localized to the medial entorhinal cortex. Both of these PNN manipulations, the TN-R knockout and Ch-ABC treatment, had a similar impact on the frequency of movement-induced theta entrainment. What this decrease in theta peak frequency could mean is unclear. Shi et al. (2019) examined theta activity and found that hippocampal theta power was increased 1 day after fear conditioning, but that treatment with Ch-ABC decreased this fear conditioning-induced theta activity. Consistent with Ch-ABC effects, when they overexpressed the PNN link protein, HAPLN1, fear conditioning-induced theta power was increased. Altogether, these hippocampal studies showed alterations in the frequency of movement-induced entrainment and deficits in memory-associated oscillations. Two of the authors, Gurevicius et al. (2004) and Christensen et al. (2021) attributed PNN manipulations to deficits in inhibition, while Shi et al. (2019) suggested that PNN removal may actually facilitate inhibition. In the visual cortex, Lensjø et al. (2017b) found that PNN removal by Ch-ABC also increased spontaneous theta power in the visual cortex. Thompson et al. (2018) found that following fear conditioning, there was an increase in coherence (phase alignment) between the theta oscillations in the secondary visual cortex and the basolateral amygdala, and that Ch-ABC treatment in the secondary visual cortex caused a decrease in coherence as well as deficits in memory retrieval. As with gamma oscillations, deficits in PV cell functioning may cause differences in evoked vs. spontaneous theta oscillations. A definitive answer as to what PNN manipulations do to a specific oscillation may be task- and brain region-specific, but it is clear that PNN removal may alter certain features of inducible oscillations, such as peak frequency and deficits in the ability to generate theta power and coherence necessary for memory recall.

#### Sharp-Wave Ripples

Yet another prominent population event that PV interneurons are tightly involved with is the hippocampal sharp-wave ripple. PV interneurons appear crucial to the synchronous temporal pacing of ripples, GABA_A_ receptor blockade abolishes ripples, and manipulations of PV interneurons alter properties such as the frequency and amplitude of ripples (Rácz et al., 2009; Schlingloff et al., 2014; Stark et al., 2014). Two studies have investigated the role of PNN removal on hippocampal sharp-wave ripples. Gurevicius et al. (2004) measured the number of hippocampal sharp-wave ripples that occurred during periods of immobility in TN-R knockout mice. They reported no changes in the frequency of ripple events when filtered between 150 and 200 Hz. It is possible that there may have been deficits in the synchronization of the underlying cells, but single unit activity was not investigated. Sun et al. (2018) found that hippocampal slices treated with either Ch-ABC or hyaluronidase had increased spontaneous ripple events that were unaltered in duration. This suggests that PNN removal may indeed alter high-frequency ripple-like oscillatory activity, potentially by decreasing inhibition that would allow for synchronous excitation to happen more readily; this synchronous excitation is thought to initiate ripples (Stark et al., 2014). Overall, PNN removal appears to alter spontaneous and evoked oscillations across a wide frequency range including theta, gamma, and sharp wave ripples in which PV interneurons have been shown to play a role.

## Impact of PNN Removal on Circuits and Implications for Memory and Behavior

PNN removal impacts the gain of PV interneurons, which has been measured by shifts in the injected current-output firing rate curves. At higher injected current levels, which PV interneurons are able to respond to with high firing rates *in vivo* (Wang et al., 2016), PNN removal reduced the firing frequency of PV interneurons (see Supplementary Table 1). What effect might this impairment at high input current have on PV interneuron computations? Faini et al. (2018) found that PNN removal in the visual cortex of mice decreased the magnitude of event-related potentials at higher contrast levels. This suggests that PNN removal may increase the gain modulation to contrast input, enhancing inhibition driven by the level of contrast, which would result in fewer neurons being responsive to the stimuli and therefore a smaller amplitude measured in the field potential. Miyata et al. (2012) reported a more depolarized resting membrane potential, as well as an increase in the action potential half-width in the visual cortex in animals with upregulated chondroitin 6-sulfation, the more immature sulfation pattern found in PNNs. These authors tested the impact of this less mature PNN network by measuring orientation tuning, optimal orientation firing rate, spontaneous firing rate, and prolonged firing following visual stimulation. They reported no change in several of these parameters but did find an increase in post-visual stimulation firing rate, which might suggest a slightly reduced inhibitory state. The results from these two studies are in conflict, with one study suggesting an increase in inhibition while the other a decrease, but the network mechanisms that mediate both may not be similar.

**Figure 3:**
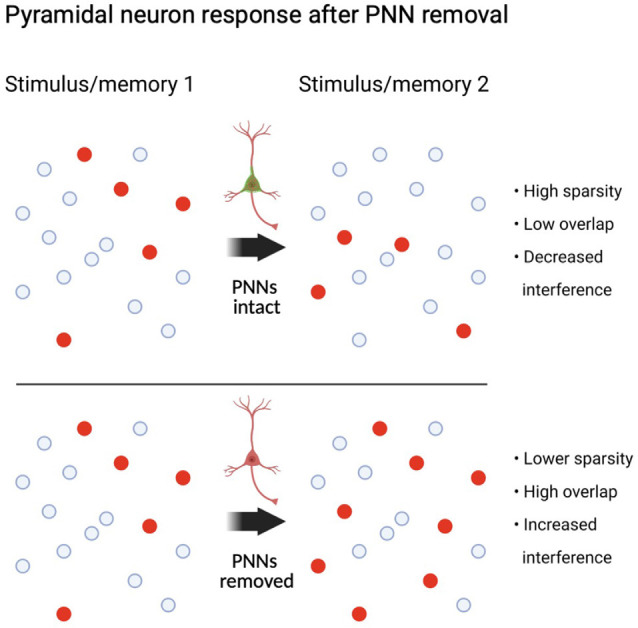
Circuit state following PV interneuron dysfunction after PNN removal. Neural assemblies originally representing a stimulus or memory may overlap when exposed to a similar stimulus or memory if PNNs are removed between the first and second exposure. Summary is based on PV interneuron specific manipulations and circuit functions (see “Role of PV Interneurons in Pattern Separation and Sparse Encoding” section). Created with BioRender.com.

The impact of PNN removal around PV interneurons produces several alterations in their electrophysiological properties (Supplementary Table 1). Collectively, 5 of 10 *in vitro* studies showed a decrease in firing rate at some injected current levels, and the only two *in vivo* reports from putative fast-spiking cells also reported decreased mean firing rate (Supplementary Table 1). *Together, this provides strong initial evidence to suggest that PNNs may increase the firing frequency of PV interneurons* (Figures 1A–C). However, there are likely to be regional differences as well as differences that depend on how PNNs are removed. PV cell excitability is of particular interest in memory and plasticity. Decreases in PV-mediated inhibition after PNN removal could *increase the size of neural assemblies*, which suggests that: (1) memory strength and memory generalization may change; and (2) that PNN removal could lead to failures in the separation of input, inducing memory interference. One intriguing new study by Christensen et al. (2021) examined how PNNs stabilize grid cell activity. They found that, when PNNs were removed, an originally stable representation by grid cells in a *familiar environment* decreased specifically when animals were exposed to a *novel environment* before their return to the familiar environment. This finding suggests that PNN removal decreased grid cell stability after novel spatial learning and is highly reminiscent of interference produced by decreases in PV interneuron activity, leading to overlapping representations. Our lab has recently made an interesting discovery that rats trained on a standard cocaine self-administration protocol, in which they learn the rule that one lever press = one cocaine infusion, do not show deficits in cue recall following PNN removal in the mPFC, as long as that rule never changes. However, if rats are re-exposed one time to a session in which they have to learn a new rule that is less predictable (average of five lever presses = one cocaine infusion), PNN removal now severely decreases cue recall the next day and for several days afterward. This suggests that exposure to a similar but new rule in the absence of PNNs degrades the *original* memory, akin to the grid cell findings (Christensen et al., 2021). These findings are consistent with the idea that, if PNN removal reduces the ability of PV interneurons to fire at higher frequencies that mediate sparse coding, it follows that suppression of PV interneurons increases the cell overlap for two stimuli that originally had separate representation in the brain (Figure 3; Agetsuma et al., 2018). In addition to altering the neural assemblies that represent an event or memory, decreased inhibition by PV interneurons could also lead to increased Hebbian plasticity, which may cause widespread alterations in overall circuit plasticity. Indeed, PV interneuron suppression alone increased auditory cortical plasticity that is similar to that found during the critical period (Cisneros-Franco and de Villers-Sidani, 2019). This may be brought about through a spreading of excitation in which cells that were originally laterally inhibited can now form new associations, and these new associations dynamically alter cortical circuits.

## Impact of Physiological Stimuli on PNNs

We have limited discussion of the studies above on the effects of PNN manipulations on the function of PV interneurons, principal neurons, and/or network properties. However, it is important to also recognize the rapidly-growing body of work supporting changes in PNNs themselves (or their key components) in response to physiological stimuli, such as stress, exercise, environmental enrichment, diet, circadian rhythms, and normal aging processes. Short term and chronic stress lead to brain region-dependent changes in PV cells and PNNs both in early life (Castillo-Gómez et al., 2017; Ueno et al., 2018; Murthy et al., 2019; Guadagno et al., 2020; Soares et al., 2020; Yu et al., 2020) and in adults (Pesarico et al., 2019; Yu et al., 2020). Exercise also alters PNNs, and the effects are dependent on the brain region examined (Smith et al., 2015; Briones et al., 2021). Several studies have shown that environmental enrichment either during early life (Carstens et al., 2016; Stamenkovic et al., 2017; O’Connor et al., 2019) or adulthood (Foscarin et al., 2011; Slaker et al., 2016) alters PNNs, as does a high-fat diet during adolescence (Reichelt et al., 2019, 2021) and adulthood (Dingess et al., 2018, 2020). Two studies have shown circadian/diurnal changes, with higher numbers or intensity of PNNs in the dark phase in rodents (Pantazopoulos et al., 2020; Harkness et al., 2021). Given that PNNs are altered in many ways throughout central nervous system development, and their maturation is brain region-specific and generally coincides with the end of critical periods of plasticity (for a recent review see Carulli and Verhaagen, 2021), it is not surprising that numerous studies have also demonstrated changes in PNNs or their composition during aging (Tanaka and Mizoguchi, 2009; Karetko-Sysa et al., 2014; Brewton et al., 2016; Foscarin et al., 2017; Richard et al., 2018; Ueno et al., 2019; Mafi et al., 2020). Overall, changes in PNNs and PV neurons after physiological stimuli appear to be specific to the physiological stimulus, brain region, and the circuits in which PNN-surrounded neurons are embedded. At present, it is difficult to make comparisons between the impact of physiological stimuli and after complete removal of PNNs because PNN removal produces abnormal circuit function, as discussed in detail above. Nevertheless, the broad picture that emerges is that PNN removal confers juvenile-like properties to PV neuron function and plasticity. However, establishing how these changes manifest within functioning circuits and in response to specific task demands need to be systematically investigated in intact systems to maximize the benefits of PNN manipulation.

## Conclusions and Future Directions

PNNs allow for the normal firing of PV interneurons, which tightly regulate pyramidal cell firing *via* dense perisomatic connections and participate in feedforward, feedback, and lateral inhibition. Removal of PNNs by the Ch-ABC enzyme or other manipulations appears to reduce the firing frequency of PV interneurons and increase the variability of PV interneuron spiking, and several major properties are likely to be altered after PNN removal. Removal of PNNs has less impact on the electrophysiological properties of principal neurons that are not enwrapped in PNNs and generally reduces LTP without altering short-term plasticity (PPR). PV interneurons coordinate long-range communication with other brain regions through the coupling of theta and gamma oscillations, which are altered when PNNs are removed. Thus, by virtue of PV properties and circuit connectivity, PNNs allow PV interneurons to play a vital role in synchronizing the output of pyramidal neurons into discrete groups of activated neurons (neural assemblies) thought to represent the coding of separate events or memories. Inhibiting PV interneuronal firing by PNN removal would therefore degrade the usually precise spatiotemporal firing patterns, producing overlapping neuronal assemblies, leading to less specificity of which assemblies represent a particular stimulus or memory. Future studies need to determine the contribution of PNN removal to PV interneuron-mediated modulation and tuning properties in other brain areas and under different modulatory states, such as attention or movement, to understand how PNN removal impacts inhibitory drive in these systems. To better understand how PNN removal impacts theta and gamma activity, future studies should investigate how PNN removal impacts behavior known to be dependent on narrowband oscillatory activity. In addition, it is important to investigate the impact of PNN removal on coherence between brain regions, phase-locking of single units to ongoing oscillations, and characterization of oscillations beyond analyzing predefined frequency ranges, such as burst detection methods, analysis of individual gamma cycle amplitudes, cycle durations, and cycle shape. We expect future *in vivo* studies in awake, behaving animals to permit a much-needed, detailed understanding of the mechanisms by which PNNs regulate the ability of their underlying PV interneurons to shape circuit function. Such an understanding has vast implications for how PNNs could be modified to optimize normal physiological functions such as learning and memory and to alleviate excitatory: inhibitory imbalances in a wide range of developmental and neurological disorders.

## Author Contributions

JW: writing original draft and editing. BS: writing original draft and editing, and funding acquistion. All authors contributed to the article and approved the submitted version.

## Conflict of Interest

The authors declare that the research was conducted in the absence of any commercial or financial relationships that could be construed as a potential conflict of interest.
