# Supplementary material for: Impact of Perineuronal Nets on Electrophysiology of Parvalbumin Interneurons, Principal Neurons, and Brain Oscillations: A Review
Source: Front Synaptic Neurosci. 2021 May 10;13:673210. doi: 10.3389/fnsyn.2021.673210 (PMC8141737; doi:10.3389/fnsyn.2021.673210)
Supplement: Supplementary file 1 [file Data_Sheet_1.PDF]

Table 1 Fast-spiking (Parvalbumin) Neuron Properties

			**Reference**	**Dityetev et al. (2007)**	**Favuzzi et al. (2017)**		
			**Brain area**	Hippocampus cultures	Hippocampus (CA1)	Hippocampus (CA1)	Hippocampus (CA1)
			**Species**	Mouse	Mouse	Mouse	Mouse
			**Age**	1-3 day mice/15-19 day culture	26-38 days	26-38 days	26-38 days
			**Sex**	Not specified	Male	Male	Both for intrinsic properties
			**PNN manipulation**	Ch-ABC in culture	Brevican (BCAN)+ or BCAN- PV+ cells	BCAN KO	BCAN knockdown in PV cells at 12 days (PV-Cre shRNA) at 30 days
			**Time(s) after PNN manipulation**	2 days *in vitro*			
			**Behavior**	None	None	Impaired working memory on alternation task; impaired short-term memory on novel object recognition task; enhanced long-term memory on novel object recognition task	Impaired short-term memory on novelty preference task
**Increase**	**Decrease**	**No Change**	**Preparation**	Culture	Slice	Slice	Slice
↑3/9	↓0/9	6/9	**Resting membrane potential**	No change	No change	No change	No change
↑2/10	↓1/10	7/10	**Input resistance**	No change	Increased	No change	Increased
↑1/2	↓0/2	1/2	**Capacitance**	No change			
↑0/9	↓5/9	4/9	**Threshold (mV)**	Decreased	No change	Decreased	Decreased; decreased rheobase
↑0/12	↓7/12	5/12	**Firing rate**	No change	Decreased maximum firing frequency in BCAN-PV+; Increased adaptation in BCAN- PV+	Decreased	Decreased maximum firing frequency; increased adaption
↑5/8	↓1/8	2/8	**Half-width (us)**	No change	Increased	Increased	Increased
↑2/6	↓1/6	3/6	**AP Amplitude or peak (mV)**	No change	No change	No change	Increased
↑1/2	↓0/2	1/2	**Rise slope (mV/ms)**				
↑0/1	↓0/1	1/1	**Decay slope (mV/ms)**				
↑0/7	↓3/7	4/7	**AHP Amplitude**	Decreased	No Change	Decreased	Decreased
↑2/6	↓0/6	4/6	**AHP Duration**		Increased	No Change	Increased
↑1/4	↓0/4	3/4	**sEPSC ampl (pA)**			No change	
↑1/5	↓2/5	2/5	**sEPSC freq (hz)**			Decreased	
↑1/3	↓0/3	2/3	**sIPSC ampl (pA)**			No change	
↑2/3	↓1/3	0/3	**sIPSC freq (hz)**			Decreased	
↑0/4	↓0/4	4/4	**mEPSC ampl (pA)**			No change	
↑1/4	↓2/4	1/4	**mEPSC freq (hz)**			Decreased	
↑0/3	↓0/3	3/3	**mIPSC ampl (pA)**			No change	
↑0/3	↓0/3	3/3	**mIPSC freq (hz)**			No change	

Table 1 Fast-spiking (Parvalbumin) Neuron Properties

Reference	Hayani et al. (2018)		Lensjo et al. (2017)	Faini et al. (2018)	Miyata et al. (2012)
**Brain area**	Hippocampus (CA2)	Hippocampus (CA2)	Visual cortex (V1)	Visual cortex (V1, L4)	Visual cortex (V1, L4)
**Species**	Mouse	Mouse	Rat	Mouse	Mouse
**Age**	Young adult (3-5 wk)	Young adult (3-5 wk)	4-6 months	Adult (>P70)	30 - 34 days
**Sex**	Not specified	Not specified	Male	Male	Not specified
**PNN manipulation**	Ch-ABC	Ch-ABC	Ch-ABC	Ch-ABC	Transgenic over-expression of C6ST-1
**Time(s) after PNN manipulation**	2 hr *in vitro*	1 week *in vivo*	3-14 days *in vivo*	2-3 days *in vivo*	
**Behavior**	None	None	Increased ocular dominance plasticity	Increased visual contrast adaptation	None
**Preparation**	Slice	Slice	*In vivo*, awake	In vivo, anesthetized and slice	Slice
**Resting membrane potential**				No Change	Increased
**Input resistance**		No change		No change	No change
**Capacitance**					
**Threshold (mV)**	No change	Decreased		No Change	
**Firing rate**	No change	No change	Decreased mean spiking activity; Increased spiking variability	No change	
**Half-width (us)**		Increased		No change	Increased
**AP Amplitude or peak (mV)**		Increased			
**Rise slope (mV/ms)**		Increased			
**Decay slope (mV/ms)**					
**AHP Amplitude**		No change			No change
**AHP Duration**		No change			No change
**sEPSC ampl (pA)**	No change	No change		Increased	
**sEPSC freq (hz)**	No change	Decreased		Increased	
**sIPSC ampl (pA)**		No change		Increased	
**sIPSC freq (hz)**		Increased		Increased	
**mEPSC ampl (pA)**	No change	No change		No change	
**mEPSC freq (hz)**	No change	Decreased		Increased	
**mIPSC ampl (pA)**	No change			No change	
**mIPSC freq (hz)**	No change			No change	

Table 1 Fast-spiking (Parvalbumin) Neuron Properties

Reference	Balmer et al. (2016)	Chu et al. (2018)	Tewari et al. (2018)	Christensen et al. (2021)
**Brain area**	Somatosensory cortex (L4-6)	Somatosensory cortex (posterior medial barrel subfield)	Primary motor and somatosensory cortex	Medial Entorhinal Cortex (MEC)
**Species**	Mouse	Mouse	Mouse	Rat
**Age**	70 days	28-52 days	6-9 weeks	3-5 months
**Sex**	Both	Both		Male
**PNN manipulation**	Ch-ABC	Ch-ABC	Ch-ABC	Ch-ABC
**Time(s) after PNN manipulation**	1 hr *in vitro*	1hr *in vitro*	45 (pretreatment before recording) or 50 min superfusion during recording *in vitro*	*5-14 days in vivo*
**Behavior**	None	None	None	Familiar and novel arena exploration
**Preparation**	Slice	Slice	Slice	*In vivo*, awake
**Resting membrane potential**	No change	Increased	Increased (pretreatment), increased (superfusion)	
**Input resistance**	No change	Decreased	No change (pretreatment), No change (superfusion)	
**Capacitance**			Increased (pretreatment), Increased (superfusion)	
**Threshold (mV)**		No Change	Decreased (pretreatment), decreased (superfusion)	
**Firing rate**	Decreased firing frequency; decreased gain, delay in firing	No change (tested up to 250 pA)	Decreased (pretreatment), decreased (superfusion)	Decreased mean spiking activity
**Half-width (us)**		Decreased		
**AP Amplitude or peak (mV)**		Decreased		
**Rise slope (mV/ms)**		No change		
**Decay slope (mV/ms)**		No change		
**AHP Amplitude**		No change		
**AHP Duration**		No change		
**sEPSC ampl (pA)**				
**sEPSC freq (hz)**		No change		
**sIPSC ampl (pA)**				
**sIPSC freq (hz)**				
**mEPSC ampl (pA)**				
**mEPSC freq (hz)**				
**mIPSC ampl (pA)**				
**mIPSC freq (hz)**				
