# Supplementary material for: Impact of Perineuronal Nets on Electrophysiology of Parvalbumin Interneurons, Principal Neurons, and Brain Oscillations: A Review
Source: Front Synaptic Neurosci. 2021 May 10;13:673210. doi: 10.3389/fnsyn.2021.673210 (PMC8141737; doi:10.3389/fnsyn.2021.673210)
Supplement: Supplementary file 2 [file Data_Sheet_2.PDF]

Table 2 Principle Neuron Properties

			**Paper Title**	**Dityatev et al. (2007)**	**Pyka et al. (2011)**	**Orlando et al. (2012)**	**Frischknecht et al. (2009)**	**Geissler et al. (2013)**
			**Brain area**	Hippocampus cultures	Hippocampus cultures	Hippocampus cultures (CA1)	Hippocampus cultures	Hippocampus cultures
			**Species**	Mouse	Rat	Mouse	Rat	Mouse
			**Age**	1-3 day/15-19 day culture	E18 cultures, grown to 13 DIV	6 day/4 week culture	E18 cultures, grown to 21 DIV	E15.5 (14 DIV and 21 DIV)
			**Sex**	Not specified	Not specified	Not specified	Not specified	Both
			**PNN manipulation**	Ch-ABC	Ch-ABC	Ch-ABC	Hyaluronidase	Tenascin-C, tenascin-R, brevican, neurocan KO in neurons or astrocytes
			**Time(s) after PNN manipulation**	48 hr *in vitro*	Added to culture every 3 days for 13 days	4 hr *in vitro*	10 min to overnight *in vitro*	
			**Behavior**	None	None	None	None	None
**Increase**	**Decrease**	**No Change**	**Preparation**	Culture	Culture	Slice	Culture	Culture
↑0/9	↓0/9	9/9	**Resting membrane potential**	No change			No change	
↑0/9	↓1/9	8/9	**Input resistance**	No change			No change	
↑0/5	↓0/5	5/5	**Capacitance**	No change	No change			
↑1/8	↓0/8	7/8	**Threshold (mV)**	No change				
↑2/12	↓3/12	7/12	**Firing rate**					
↑1/9	↓0/9	8/9	**Half-width (us)**	No change			No change	
↑0/7	↓0/7	7/7	**AP Amplitude (mV)**	No change			No change	
↑0/3	↓0/3	3/3	**AP peak (mV)**					
↑0/4	↓0/4	4/4	**Rise slope (mV/ms)**					
↑1/4	↓0/4	3/4	**Decay slope (mV/ms)**					
↑0/4	↓0/4	4/4	**AHP Amplitude**	No change				
↑0/3	↓0/3	3/3	**AHP Duration**					
↑0/3	↓0/3	3/3	**sEPSC ampl (pA)**					
↑0/4	↓1/4	3/4	**sEPSC freq (Hz)**					
↑1/6	↓0/6	5/6	**sIPSC ampl (pA)**					
↑2/5	↓0/5	3/5	**sIPSC freq (Hz)**					
↑0/6	↓1/6	5/6	**mEPSC ampl (pA)**		Decreased	No change; no change in rise or decay time	No change	No change
↑1/6	↓1/6	4/6	**mEPSC freq (Hz)**		No change	No change	No change	Decreased
↑1/9	↓0/9	8/9	**mIPSC ampl (pA)**		No change			No change
↑3/9	↓4/9	2/9	**mIPSC freq (Hz)**		No change			Decreased
			**Other**		No change in voltage-activated sodium or potassium currents after Ch-ABC; decreased amplitude of glutamate-induced mEPSCs	Ch-ABC increased spine remodeling locally and independently of PNNs	Decreased PPD; PPF after hyaluronidase; higher increase by blocking AMPAR desensitization and lower by immobilizing AMPARs with antibody cross-linking; greater AMPAR diffusion after hyaluronidase	PNN-surrounded neurons have same differences as non-PNN-surrounded neurons for mEPSCs and mIPSCs

Table 2 Principle Neuron Properties

Paper Title	Khoo et al. (2019)	Kochlamazashvili et al. (2010)	Shi et al. (2019)	Saghatelyan et al. (2001)	Brakebusch et al. (2002)	Carstens et al. (2016)
**Brain area**	Hippocampus (CA1)	Hippocampus (CA1)	Hippocampus (CA1)	Hippocampus (CA1)	Hippocampus (CA1)	Hippocampus (CA2)
**Species**	Mouse	Mouse	Mouse	Mouse	Mouse	Mouse
**Age**	Adult (P56-84)	2-3 months	Adult (3 months)	13-20 days and 3 month	Adult	14-18 days
**Sex**	Male	Both	not specified	Not specified	Male	Male
**PNN manipulation**	Ch-ABC	Hyaluronidase	Ch-ABC, brevican (BCAN) knockdown (shBCAN), Hapln1 over-expression	Tenascin-R KO (TN-R KO)	BCAN KO	Ch-ABC
**Time(s) after PNN manipulation**	2hr *in vitro*	2 hr *in vitro* or 24 hr *in vivo* (behavior)	1 day *in vivo*			2 hr *in vitro*
**Behavior**	None	Reduced retrieval but not acquisition of contextual fear conditioning (only males tested)	Contextual fear memory decreased after Ch-ABC in CA1 and anterior cingulate cortex; Hapln1 overexpression in anterior cingulate cortex increased fear	None	No change in active avoidance in shuttle box; no change in gross sensory function or reflexes; increase in forelimb grip strength; No change in motor behavior, four-holeboard, plus maze, active-avoidance; decrease in probe trial crossings	None
**Preparation**	Slice	Slice	Slice	Slice	Slice	Slice
**Resting membrane potential**		No change				No change
**Input resistance**		No change				Decreased
**Capacitance**						No change
**Threshold (mV)**		No change				No change
**Firing rate**	No change					
**Half-width (us)**		No change				
**AP Amplitude (mV)**		No change				No change
**AP peak (mV)**						
**Rise slope (mV/ms)**						No change
**Decay slope (mV/ms)**						No change
**AHP Amplitude**						
**AHP Duration**						
**sEPSC ampl (pA)**						
**sEPSC freq (Hz)**						
**sIPSC ampl (pA)**			No change after Ch-ABC; Increased in shBCAN		No change	
**sIPSC freq (Hz)**			Increased after Ch-ABC and in shBCAN, decreased by Hapln1 overexpression		No change	
**mEPSC ampl (pA)**				No change		
**mEPSC freq (Hz)**				Increased		
**mIPSC ampl (pA)**	No change		No change after Ch-ABC; increased in shBCAN	No change		
**mIPSC freq (Hz)**	Decreased		Increased in shBCAN and decreased in Hapln1 overexpression	Increased		
**Other**	Stimulated EPSP/IPSP ratio increased due to a decrease in stimulated IPSP amplitude; no change in PPF	No change in spike adaptation	Paired pulse ratio (PPR) increased in both Ch-ABC and shBCAN	Decreased amplitude of perisomatic unitary currents; no change in PPF; basal synaptic transmission higher in TN-R KO	No change in rise or decay time of sIPSCs; no change in synaptic AMPA or NMDA currents	Decay time for intrinsic properties, no change; in presence of bicuculline, no change in PPF or AMPA/NMDA ratio

Table 2 Principle Neuron Properties

Paper Title	Hayani et al. (2018)		Shah and Lodge et al. (2013)	Lensjo et al. (2017)	Faini et al. (2018)	Chu et al. (2018)	Tewari et al. (2018)
**Brain area**	Hippocampus (CA2)	Hippocampus (CA2)	Ventral hippocampus	Visual cortex (V1)	Visual cortex (V1, L4)	Somatosensory cortex (post barrel medial subfield	Primary motor and somatosensory cortex
**Species**	Mouse	Mouse	Rat	Rat	Mouse	Mouse	Mouse
**Age**	Young adult (3-5 wk)	Young adult (3-5 wk)	Adult	Adult (4-6 months)	Adult (>P70)	28-52 days	6-9 weeks
**Sex**	Not specified	Not specified	Male	Male	Male	Both	Both
**PNN manipulation**	Ch-ABC	Ch-ABC	Ch-ABC	Ch-ABC	Ch-ABC	Ch-ABC	Ch-Abc
**Time(s) after PNN manipulation**	2 hr *in vitro*	7 days *in vivo*	7 days or more *in vivo*	3-14 days *in vivo*	2-3 days *in vivo*	1 hr *in vitro*	45 pretreatment or 50 min superfusion during recording *in vitro*
**Behavior**	None	None	Increased locomotor response to amphetamine	Increased ocular dominance plasticity	None	None	None
**Preparation**	Slice	Slice	*In vivo*, anesthetized	*In vivo*, awake	*In vivo*, anesthetized and slice	Slice	Slice
**Resting membrane potential**					No change	No change	No change (pretreatment), Increased (superfusion)
**Input resistance**	No change	No change			No change	No change	No change (pretreatment), No change (superfusion)
**Capacitance**							No change (pretreatment), No change (superfusion)
**Threshold (mV)**	No change	No change			No Change	No change	
**Firing rate**	No change	No change	Increased percent of cells firing at higher frequency	No change, but increase in spiking variability	No change	No change	Increased (pretreatment), not reported (superfusion)
**Half-width (us)**		No change			No change	No change	
**AP Amplitude (mV)**		No change				No change	
**AP peak (mV)**					No change	No change	
**Rise slope (mV/ms)**		No change				No change	
**Decay slope (mV/ms)**						No change	
**AHP Amplitude**		No change				No change	
**AHP Duration**		No change				No change	
**sEPSC ampl (pA)**	No change	No change			No change		
**sEPSC freq (Hz)**	No change	Decrease			No change	No change	
**sIPSC ampl (pA)**		No change			No change		
**sIPSC freq (Hz)**		No change, but faster decay time			No change		
**mEPSC ampl (pA)**	No change						
**mEPSC freq (Hz)**	No change						
**mIPSC ampl (pA)**	No change						
**mIPSC freq (Hz)**	No change						
**Other**		Decreased AP latency		Trend for lower evoked activity in attentive state	Feed-forward inhibition (evoked IPSC) from thalamic optical stimulation is increased at 1.5x the threshold stimulation in Ch-ABC animals; attenuated by monocular deprivation (MD)		

Table 2 Principle Neuron Properties

Paper Title	Slaker et al. (2015)	Christensen et al. (2021)	Blosa et al. (2015)	Balmer et al. (2016)	Hirono et al. (2018)	Edamatsu et al. (2018)	Carulli et al. (2020)
**Brain area**	Medial prefrontal cortex	Medial entorhinal cortex (putative stellate cells)	Medial nucleus of the trapezoid body(MNTB)	Medial nucleus of the trapezoid body(MNTB)	Deep cerebellar nucleus (DCN)	Deep cerebellar nucleus (DCN)	Deep cerebellar nucleus (DCN)
**Species**	Rat	Rat	Mouse	Mouse	Mouse	Mouse	Mouse
**Age**	Adult	Adult	Young adult (23-31 days)	21 - 28 days	Days 18-30	Day 14	6-8 weeks
**Sex**	Male	Male	Both	Both	Male	Both	Male
**PNN manipulation**	Ch-ABC	Ch-ABC	BCAN KO	Ch-ABC	Ch-ABC	Hapln4/Bral2 KO	Viral expression of Ch-ABC
**Time(s) after PNN manipulation**	9 days *in vivo*	5-14 days in-vivo		1 hr *in vitro*	3-6 hr *in vitro* or 4-6 days *in vivo* for slice recording; 6 days for *in vivo* recording		3 weeks *in vivo*
**Behavior**	Decreased cocaine-induced conditioned place preference	Familiar and novel arena exploration	None	None	Eyeblink conditioning acquisition increased (8-12 week old mice)	None	Eyeblink conditioning acquisition increased; retention of conditioning decreased
**Preparation**	Slice	*In vivo*, awake	*In vivo,* anesthetized	Slice	*In vivo*, awake (for eye blink conditioning) or slice	Slice	*In vivo*, awake
**Resting membrane potential**				No change		No change	
**Input resistance**				No change; no change in rectification			
**Capacitance**						No change	
**Threshold (mV)**				Increased			
**Firing rate**	Increased	Decreaseed mean firing rate, decreased max in field firing rate	Decreased sound-evoked firing rate	Decreased firing frequency, lower gain			
**Half-width (us)**			Increased	No change		No change	
**AP Amplitude (mV)**				No change			
**AP peak (mV)**						No change	
**Rise slope (mV/ms)**				No change			
**Decay slope (mV/ms)**			Increased	No change			
**AHP Amplitude**				No change			
**AHP Duration**				No change			
**sEPSC ampl (pA)**							
**sEPSC freq (Hz)**							
**sIPSC ampl (pA)**					Increased	No change	
**sIPSC freq (Hz)**					Increased		
**mEPSC ampl (pA)**							
**mEPSC freq (Hz)**							
**mIPSC ampl (pA)**	No change				No change	No change	
**mIPSC freq (Hz)**	Decreased				Increased		
**Other**		Grid cell stability decreased, further decline noticed following exposure to novel environment	No change in spontaneous discharge activity; reliability of sound-evoked activity normal but speed of AP slower due to broadening of presynaptic AP peak and slower and broader post-synapse EPSP at calyx of Held synapse	Able to fire up to 1000 Hz after Ch-ABC	Increased amplitude of evoked IPSCs (Purkinje cell stimulation); increased depression of eIPSCs by stimulation train; no change in evoked IPSCs; rebound firing frequency higher; decrease in PPR	Larger potassium current in KO mice; no change in input-output curve; decreased evoked IPSC amplitudes (Purkinje cell stimulation); no change in PPR; no change in evoked EPSPs (mossy fiber stimulation)	Decreased spontaneous firing frequency of DCN neurons (anterior interpositus nucleus neurons)
