# Supplementary material for: Impact of Perineuronal Nets on Electrophysiology of Parvalbumin Interneurons, Principal Neurons, and Brain Oscillations: A Review
Source: Front Synaptic Neurosci. 2021 May 10;13:673210. doi: 10.3389/fnsyn.2021.673210 (PMC8141737; doi:10.3389/fnsyn.2021.673210)
Supplement: Supplementary file 3 [file Data_Sheet_3.PDF]

Table 3 Plasticity Properties

Paper Title	Bikbaev et al. (2015)	Gottschling et al. (2019)	Frischknecht et al. (2009)	Jansen et al. (2017)	Riga et al. (2017)	Khoo et al. (2019)
**Brain Area**	Hippocampus cultures on microelectrode array	Hippocampus neuron+astrocyte co-cultures on multielectrode array	Hippocampus cultures	Hippocampus (dentate gyrus) stimulation in perforant pathway	Hippocampus (CA1) stimulation in Schaffer collaterals	Hippocampus (CA1) stimulation in Schaffer collaterals
**Species**	Rat	Mouse	Rat	Mouse	Rat	Mouse
**Age**	E18 grown 4 wks DIV28	Embryonic 15.5 (14 DIV and 21 DIV)	E18 cultures, grown to 21 DIV	8-9 weeks	6-8 weeks	Adult P56-84
**Sex**	Not specified	Both	Not specified	Not specified	Male	Male
**PNN manipulation**	Hyaluronidase	Tenascin-C, tenascin-R, brevican, neurocan KO in neurons or astrocytes	Hyaluronidase	Tenascin-C, tenascin-R, brevican, neurocan KO mice	Ch-ABC	Ch-ABC
**Time(s) after PNN manipulation**	1 hr *in vitro*		10 min to overnight *in vitro*		2 weeks *in vivo*	2 hr *in vitro*
**Behavior**	None	None	None	None	No effect on object place recognition task; decreased social recognition, but restored social defeat stress-induced impairment on social recognition memory and object place recognition	None
**Preparation**	Culture	Cultured neurons and astrocytes	Culture	*In vivo*, awake	Slice	Slice
**LTP**				High-frequency stimulation induced initial decrease followed by delayed LTP in KO mice for fEPSP and population spike amplitude; both groups show LTP when given weak high-frequency stimulation	No change (fEPSP)	
**LTD**				Short-term depression larger and longer lasting in KO mice for fEPSPs and population spike amplitude; no LTD observed in WT or KO mice		Produced LTD after Ch-ABC, which was not observed in controls; no change in basal fEPSP
**Paired pulse ratio and other outcomes**	Increased spiking and bursting rate;<br>no change in mean burst duration, spikes/burst or intra-burst inter-spike interval;<br>increased network burst rate and duration;<br>no change in bursting synchronization	Spontaneous activity at 14 and 21 DIV in quadruple knockout in co-cultures of both neurons and astrocytes: increased number and spikes and spike frequency; increased number of bursts; increased spike frequency in bursts; increased percentage of spikes in burst, and at 21 DIV: increased burst duration. Overall, increased network activity	Decreased PPD; more neurons show PPF after hyaluronidase; this increase was higher by blocking AMPAR desensitization and lower by immobilizing AMPARs with antibody cross-linking	LTP elicited by weak stimulation NMDA-dependent in WT but not KO mice	No change in amplitude of sIPSCs; Ch-ABC restored frequency of sIPSCs and LTP in social defeat stress-induced rats	No change in PPF; NMDA receptor antagonist and GABA agonist blocked LTD after Ch-ABC

Table 3 Plasticity Properties

Paper Title	Shi et al. (2019)	Bukalo et al. (2001)	Kochlamazashvili et al. (2010)	Saghatelyan et al. (2001)	Zhou et al. (2001)	Brakebusch et al. (2002)
Brain Area	Hippocampus CA1 stimulation in Schaffer collaterals (recording); anterior cingulate cortex (behavior)	Hippocampus (CA1) stimulation in Schaffer collaterals	Hippocampus (CA1) stimulation in Schaffer collaterals	Hippocampus (CA1) stimulation in Schaffer collaterals	Hippocampus (CA1) stimulation in Schaffer collaterals	Hippocampus (CA1) stimulation in Schaffer collaterals
Species	Mouse	Mouse	Mouse	Mouse	Mouse	Mouse
Age	Adult (3 months)	Adult (3-4 months)	2-3 months	13-20 days and 3 month	Adult	Adult
Sex	Not specified	Not specified	Both	Not specified	Male	Male
PNN manipulation	Ch-ABC and shBCAN	Ch-ABC and TN-R KO	Hyaluronidase	Tenascin-R knockout (TN-R KO)	Neurocan knockout	Brevican knockout (BCAN KO)
Time(s) after PNN manipulation	24 hr *in vivo*	2 hr *in vitro*	2 hr *in vitro* or 24 hr *in vivo* (behavior)			
Behavior	Contextual fear memory consolidation decreased after Ch-ABC or shBCAN in CA1; Ch-ABC in anterior cingulate cortex reduced recall of fear memory at 1 week but not 3 weeks; Hapln1 overexpression in anterior cingulate cortex promoted fear memory retention; shBCAN in CA1reduced acquisition of fear	None	Reduced retrieval but not acquisition of contextual fear conditioning (only males tested)	None	None	No change in active avoidance in shuttle box; no change in gross sensory function or reflexes; increase in forelimb grip strength; No change in motor behavior, four-holeboard, plus maze, active-avoidance; decrease in probe trial crossings
Preparation	Slice	Slice	Slice	Slice	Slice	Slice
LTP	Decreased (fEPSP) by Ch-ABC, rescued by picrotoxin	Decreased (fEPSP) by Ch-ABC and TN-R KO mice; no further reduction in TN-R KO + Ch-ABC	Decreased (fEPSP) in a stimulation-dependent manner (addition of hyaluronic acid restored LTP); decrease in LTP due to postsynaptic voltage-dependent calcium channels	Decreased (fEPSP) in 13-20 and 3 month	Decreased (fEPSP)	Decreased (fEPSP) in BCAN KO mice and with BCAN antibody
LTD	Theta burst stimulation produced IPSC depression (GABA LTD) in controls but potentiation in Ch-ABC animals in presence of glutamate blockers	Transient potentiation not different for the NMDA component in either TN-R KO or Ch-ABC mice;<br>short-term depression reduced in TN-R KO but no change after Ch-ABC;LTD reduced in Ch-ABC but not TN-R KO mice (fEPSP)				
Paired pulse ratio and other outcomes	Ch-ABC and shBCAN in CA1 or anterior cingulate cortex reduced and Hapln1 overexpression enhanced apparent reconsolidation; Ch-ABC increased spontaneous IPSCs; no changes in PPR of IPSCs during baseline, but stimulation increased PPR after Ch-ABC and shBCAN.	No change in basal synaptic transmission in TN-R plus/minus Ch-ABC<br>PPF: no change in either group	No change in PPF<br><br>No change in basal fEPSPs	No change in PPF; Basal synaptic transmission higher in TN-R KO	No change in input-output curve; no change in PPF	No change in PPF;<br>No change in PPD

Table 3 Plasticity Properties

**Paper Title**	**Carstens et al. (2016)**	**Hirono et al. (2018)**	**Edamatsu et al. (2018)**	**Romberg et al. (2013)**
Brain Area	Hippocampus (CA2)	Deep cerebellar nucleus (DCN)	Deep cerebellar nucleus (DCN)	Perirhinal cortex stimulation in lateral entorhinal cortex
Species	Mouse	Mouse	Mouse	Mouse
Age	14-18 days	Days 18-30	Day 14	3-4 months
Sex	Male	Male	Both	Male
PNN manipulation	Ch-ABC	Ch-ABC	Hapln4/Bral2 KO	Crtl1 KO mice; Crtl1 over expressing; Ch-ABC
Time(s) after PNN manipulation	2 hr *in vitro*	3-6 hr *in vitro* or 4-6 days *in vivo* for slice recording; 6 days for *in vivo* recording	None	7 days (behavior); not stated for LTD
Behavior	None	Eyeblink conditioning acquisition increased (8-12 week old mice)	None	Ch-ABC and Crtl1 KO: Increased novel object recognition memory (24 and 48 hr later), no greater effect of Ch-ABC in Crtl1 KO mice; effect of Ch-ABC lasts 3 weeks, but not 6 or 8 weeks
Preparation	Slice	Slice	Slice	Slice
LTP				
LTD				Increased (fEPSP) in Ch-ABC and Crtl1 KO mice
Paired pulse ratio and other outcomes	In presence of bicuculline, no change in PPF or AMPA/NMDA ratio	Increased amplitude of evoked IPSCs (Purkinje cell stimulation); increased depression of eIPSCs by stimulation train; no change in evoked IPSCs; rebound firing frequency higher; increase in PPD (increased paired-pulse IPSCs)	No change in PPR; no change in evoked EPSPs (mossy fiber stimulation)	In Ch-ABC and Crtl1 KO mice, greater basal fEPSPs, decrease in PPF in Crtl1 KO and after Ch-ABC
