# Supplementary material for: Impact of Perineuronal Nets on Electrophysiology of Parvalbumin Interneurons, Principal Neurons, and Brain Oscillations: A Review
Source: Front Synaptic Neurosci. 2021 May 10;13:673210. doi: 10.3389/fnsyn.2021.673210 (PMC8141737; doi:10.3389/fnsyn.2021.673210)
Supplement: Supplementary file 4 [file Data_Sheet_4.PDF]

Table 4 Oscillations

Paper Title	Sun 2018	Gurevicius 2004	Shi 2018	Lensjo 2017	Hou 2017	Faini 2018
**Brain Area**	Hippocampus (CA1)	Hippocampus (CA1, dentate gyrus (DG)	Hippocampus (CA1)	Visual cortex (V1, L 2/3)	Visual cortex (V1)	Visual cortex (V1)
**Species**	Mouse	Mouse	Mouse	Rats	Mouse	Mouse
**Age**	6-8 weeks	Adult (9-11 months)	Adult (3 months)	Adult (4-6 months)	28-30 days	Adult (>P70)
**Sex**	Both	Male	Not specified	Male	Both	Male
**PNN model**	Ch-ABC *in vitro* Hyaluronidase *in vitro*	Tenascin-R KO	Ch-ABC and shBCAN	Ch-ABC	CSGalNAcT1 (key CS synthesizing enzyme)	Ch-ABC
**Time since treatment**	2 hr		1 day	3-14 days		2-3 days
**Preparation**	*In vitro*	*In vivo*, awake freely behaving	*In vivo*, CA1	*In vivo*, awake freely behaving		*In vivo*, anesthetized
**Behavior/Stimulus**	Spontaneous occurrence	Auditory gating paradigm (2 sharp clicks separated by interval)	Contextual fear conditioning	Drifting gratings, monocular deprivation		Visually-evoked response to different contrast levels and changes in power spectrum.
**Outcome**	Increased SWR occurrence frequency; no change in duration; increased SWR occurrence frequency; no change in duration	Same number of SWRs, increase by 100% of gamma power; decreased frequency of theta, same peak theta power; EEG power increased at 2-6, 14-20, and 20-40 Hz range; increased amplitude of auditory-evoked potentials	In either CA1 or ACC: decreased remote fear memory expression and decreased reconsolidation; theta oscillations increased 24 hr after fear conditioning. Ch-ABC decreased fear conditioning-induced theta activity; overexpression of HAPLN1 increased theta power	Increased spontaneous power in theta and low-gamma; no change in visual stimulus induced power; Ch-ABC increased ocular dominance plasticity; increased gamma power (40-55 Hz) immediately after MD, which disappeared after 1 hr	Calcium imaging showed reduced response to visual stimuli in PV but not non-PV cells	Decreased visually-evoked response amplitude to higher contrast; Ch-ABC increased resting state gamma (40-80Hz) power as well as during 100% contrast checkerboard stimulation (broadband increase)

Table 4 Oscillations

Paper Title	Thompson 2018	Cabungcal 2013	Steullet 2014	Carcellar 2020	Christensen 2021
Brain Area	Visual cortex (V2L for Ch-ABC + record) and BLA (record only)	Anterior cingulate cortex (ACC)	Anterior cingulate cortex (ACC)	Medial prefrontal cortex (mPFC)	Medial entorhinal cortex (MEC), hippocampus (CA1)
Species	rats	Gclm KO mouse	Mouse	Mouse	rats
Age	Not specificied	Adult (90 days)	Adult (3-5 months)	Adult (3 months)	3-8 months
Sex	Male	Not specified	Not specified	Male	Male
PNN model	Ch-ABC	Ch-ABC	Ch-ABC	Ch-ABC	Ch-ABC
Time since treatment	> 7 days	Not specified	3 days	4 days	3-14 days
Preparation	*In vivo*, freely behaving	*In vitro*	*In vitro*	*In vivo*, anesthetized	*In vivo*, freely behaving
Behavior/Stimulus	Fear conditioning and context re-exposure	carbechol, kainate, quinpirole	carbechol, kainate, quinpirole	Tail pinch	Arena exploration familiar and novel
Outcome	Decreased theta coherence during memory retrieval; no impact on cue-evoked response	Increased power of high-frequency oscillations-beta/gamma	Increased power of high-frequency oscillations	Decreased high-frequency >70Hz power; decreased modulation index for theta-gamma coupling	Theta power increased and peak frequency decreased; theta peak frequency decreased during initial novel exploration but continued to increase during prolonged exploration-more so in the control group; this effect not mediated by running speed modulation; hippocampal theta power increased, although treatment localized to MEC
